# Differences in the dynamics of community disaster resilience across the globe

**DOI:** 10.1038/s41598-021-96763-0

**Published:** 2021-09-02

**Authors:** Stefan Hochrainer-Stigler, Stefan Velev, Finn Laurien, Karen Campbell, Jeffrey Czajkowski, Adriana Keating, Reinhard Mechler

**Affiliations:** 1 IIASA-International Institute for Applied Systems Analysis, Laxenburg, Austria; 2 Wharton Risk Management and Decision Processes Center, Philadelphia, USA; 3 IHS Markit, Philadelphia, USA; 4 Center for Insurance Policy and Research, National Association of Insurance Commissioners, Kansas, USA

**Keywords:** Environmental sciences, Environmental impact, Natural hazards, Environmental impact, Sustainability

## Abstract

The consideration of disaster resilience as a multidimensional concept provides a viable and promising way forward for reducing risk and minimizing impacts today and in the future. What is missing is the understanding of the actual dynamics of resilience over time based on empirical evidence. This empirical understanding requires a consistent measure of resilience. To that end, a Technical Resilience Grading Standard for community flood resilience, was applied in a longitudinal study from 2016 to 2018 in 68 communities across the globe. We analyse the dynamics of disaster resilience using an advanced boosted regression tree modelling framework. The main outcome of our analysis is twofold: first, we found empirical evidence that the dynamics of resilience build on a typology of communities and that different community clusters experience different dynamics; and second, the dynamics of resilience follows transitional behaviour rather than a linear or continuous process. These are empirical insights that can provide ways forward, theoretically as well as practically, in the understanding of resilience as well as in regard to effective policy guidance to enhance disaster resilience.

## Introduction

Floods are one of the major hazards around the world: more than 90% of the total 7200 events recorded in EM-DAT in the last two decades were climate-related, with floods being the most frequent type of disaster, comprising about 43% of total events. Over the same time period, floods caused more than 661 billion USD of losses (around 23% of total losses from all events) and around 124,088 fatalities (around 11% of all fatalities)^1^. Dealing with such disasters is a challenge for many reasons but especially because impacts are often disproportionally borne by the poor^2,3^. This is particularly troubling since reduction of poverty is a key dimension for the efficient and sustainable management of flood risk. Concerningly, the number of disaster events and the amounts of both total and insured disaster losses have all been increasing over time^4^. The current understanding is that the increases in disaster risk has been largely driven by the extent to which humans and assets are increasingly exposed to natural hazards and the extent to which they are resilient to them^5,6^. While it is now generally acknowledged that the actual occurrence of a natural hazard can be influenced by climate change^7^, the level of impact is largely influenced by non-climatic factors^5^. Hence, a deeper understanding of the factors underlying and causing disaster risks is becoming increasingly imperative.

The consideration of resilience as a multidimensional concept provides a viable and promising way forward for achieving this understanding, as well as for identifying the most promising options for reducing risk today and in the future^8–10^. The Sendai Framework for Risk Reduction and the Sustainable Development Goals have both integrated this need for resilience in their goals^2,11^. However, while the resilience discussion has progressed immensely over the last years^12^ and it is understood that resilience may vary spatially, temporally and over different sub-groups of the exposed population (e.g. the distributional aspects), the actual, empiral, understanding of the dynamics (here understood as temporal or spatial changes^13^) of resilience over time remains still limited^14^. Additionally, apart from a few exceptional examples that focus on specific local regions to assess the dynamics of resilience, empirical analysis of the possible dynamics from a global perspective at the local level is yet to be undertaken^15^. There are a few major challenges that can explain the scarcity of much needed empirical research on the dynamics of resilience. One of the largest challenges is to establish a standardized resilience measurement approach that can explicitly take into account the distributional aspects of resilience across different dimensions, while still being comparable globally.

Although myriad of definitions and conceptualizations about resilience have been proposed by the academy, multilateral organizations, development agencies, nongovernmental organizations and others^16^, standardized measurements and assessments of resilience which can be used across the globe are still lacking^17^. This is problematic because it makes it impossible to compare resilience levels between risk bearers (e.g. households, communities or countries), to track progress over time and to learn and establish best practices. To fill this gap the Flood Resilience Measurement for Communities and Technical Resilience Grading Standard (TResGS)—was developed by the Zurich Flood Resilience Alliance (see Keating et al.^18^ and Campbell et al.^19^). The FRMC is a consistent benchmark against which to quantify flood resilience at the community level. In addition to this consistent benchmark, an impact assessment is conducted for measuring the revealed resilience of the disaster (called post-flood event study).

The definition of resilience underlying the FRMC is: “The ability of a system, community, or society to pursue its social, ecological, and economic development and growth objectives, while managing its disaster risk over time in a mutually reinforcing way”^18^. Central to this concept is the distinction between five key capitals which holistically make up the socio-economic system of communities. Across these capitals are 88 indicators of sources of resilience (sources) as they contribute to a community’s capacity to reduce risk, prepare for, withstand and recover better from a flood disaster. These sources were measured at baseline and endline of the project, and a post-flood study impact assessment taken after a flood event within selected communities across the globe. How resilience changes over time (e.g. due to hazard events or interventions) is what we will call the ‘dynamics of resilience.’ These dynamics include interactions (such as substitution and complementation) among the sources of resilience within each of the capitals at different times and in different regions^13^.

The main outcome of our analysis is twofold: first, we find empirical evidence that the dynamics of disaster resilience shows similar patterns based on the typology (i.e. specific characteristics) of communities and second, the dynamics of resilience can also follow a non-linear behaviour pattern rather than a linear and continuous process. These are important empirical insights that have good theoretical underpinnings but to date have not been empirically tested^20^. We start our discussion with the resilience measurement and data gathering process.

## Resilience measurement and data gathering

To date, there is much theory about what contributes to resilience and how disaster impacts and resilience capacity are related, however, empirical studies that analyze these dynamics on the ground in multiple and diverse contexts are still an underdeveloped topic^12,18^. One reason for that is the need to have a standardized resilience measurement framework to collect the required data across the globe. We describe the developed framework and tool that can be used for such an analysis, called *Flood Resilience Measurement Approach.* The theoretical conceptualization and framework of the Flood Resilience Measurement for Communities and Technical Resilience Grading Standard are extensively discussed in Keating et al.^18^. The central concept is around key community capitals which is based on and further developed within the sustainable livelihood framework (Ashley and Carney^21^). As our definition of resilience indicates, the focus is on resilience-wellbeing nexus, rather than the more prominent emphasis on rapid recovery in other resilience definitions (see for example the discussion in Hassan and Mahmoud^22^). In a nutshell the FRMC approach measures the pre-flood characteristics (called ‘sources of resilience’) that contribute to a community’s capacity to reduce risk, prepare for, withstand and recover *better* from a flood disaster. A comprehensive set of 88 sources of resilience across five capitals (human, physical, social, natural and financial) was developed that can ultimately be quantified through a systematic, multi-option, and flexible data collection methodology and grading process.

**Figure 1:**
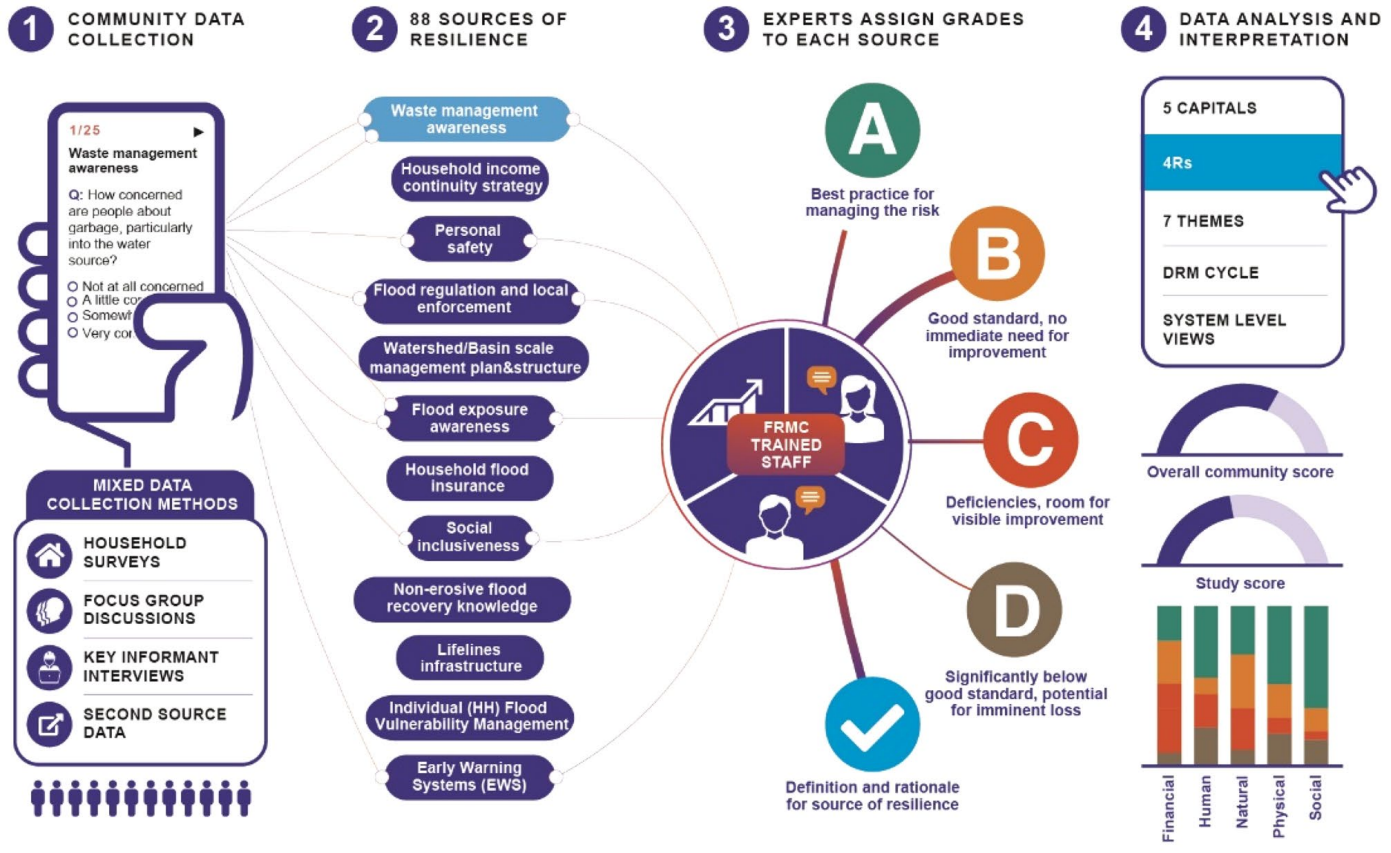
FRMT data implementation process. [Source: (Laurien et al.^14^)].

The underlying framework was designed by members of the Zurich Flood Resilience Alliance comprising of representatives from the NGO sector, academia, and risk engineering expertise. In this way, the framework balances flexibility so that it can be implemented collaboratively by community stakeholders, while maintaining enough consistency to get comparable data with high standard. The outcome of the framework design resulted in the operationalized tool (FRMT)—an integrated, web-based and mobile device platform for creating questionnaires based on a flexible combination of data collection methods for each of the 88 indicators (sources of resilience), assigning data collection work, collecting data, undertaking grading ranging from A to D for each source, generating outputs, and storing data on a (protected) central database (see Fig. 1).

An initial empirical analysis of the FRMT can be found in Campbell et al.^19^ and an analysis of the resilience characteristics can be found in Laurien et al.^14^. In Hochrainer-Stigler et al.^23^ and Laurien et al.^14^ the internal consistency of the framework and the inter-grader reliability was tested. Here, the characteristics of resilience are linked to disaster impacts (post-event study) to assess the dynamics between two time periods (baseline and endline measures), as well as for communities that experienced a flood event over the study period. It should be mentioned that the framework and analysis presented here are complementary to rather than a replacement for current vulnerability and capacity assessments (for a detailed discussion of resilience and vulnerability see Keating et al.^18^ and for a vulnerability related analysis see for example Kreibich et al.^24^). As mentioned, resilience is a latent construct until an event happens. The sources of resilience were designed to provide a measure of the capacities in a community to be resilient in the event of a flood. The post-event studies measure a community’s actual resilience in the event of a flood. As this paper is the first paper to present findings from the post-event studies, we give some additional information of the structure of the variables assessed.

The post-event studies are designed to measure the impact of flood events in terms of revealed resilience (level of losses and time to recovery). Similar to the baseline (BL) and repeated baseline (endline EL) measures of the sources of resilience, the post-event study is also a holistic measure to evaluate the impacts and effects across the community functions. Specifically, community post-event studies are structured around seven themes and consists of three control variables, 19 impact variables and seven action variables (see Keating et al.^18^). It is a measure of revealed flood resilience in the case a flood event occurs in the community. Three types of variables are considered in the post-event studies: control variables that gauge the severity of the flood itself, impact variables that measure direct and indirect damages, and action variables that measure actions taken prior, during or after the flood event to mitigate the losses (the specific questions asked in the baseline and post-event study can be found in Keating et al.^18^).

### Baseline, post-event and endline data

Trained expert assessors review the on-the-ground data collected from surveys, interviews or third-party sources and assign a grade from A to D for each indicator (i.e., source of resilience or actual resilience indicators). This was done for two time periods, called baseline (BL) and endline (EL). For those communities which experienced a flood event an additional post-event study (PE) was performed. Hence, the final database for our analysis consisted of all 88 graded sources of resilience for the BL and EL studies, as well as the 26 post-event (outcome) indicators (plus 3 ungraded indicators) in those communities which experienced a flood event between the two data collection periods (i.e. between 2016 and 2018). The 88 sources are also summarized (averaged) into the five capitals (for more details see also Keating et al.^18^). Summarizing, in each community where the FRMT was implemented the following data was collected (Table 1).

**Table 1:** Datasets over time periods and community characteristics used for analysis. Adapted from^14^.


Dataset	Description	Number of communities
Baseline (BL)	Baseline measurement of 88 assigned grades of ‘sources of resilience’ and ‘essential’ community characteristic data	118
Post-event (PE)	Post-flood impact data based on 26 outcome indicators (if a flood occurred in the community), including flood severity information	17
Endline (EL)	Endline measurement which is a repeat of the baseline exercise conducted one to 2 years after the baseline data measurement	68
Community characteristics (CC)	Qualitative data collected on interventions implemented in the community, including timeline of interventions and flood (if it occurred), as well as community, regional, and country characteristics (in total 25 CC)	118

Communities were selected based on a set of criteria including the need for external support, exposure to flood risk, location of communities within broader river basins as well as willingness to be part of the program (see Supplementary A for more details). It should be mentioned that while the criteria for selecting communities across countries were similar, the communities vary in regards to several key community characteristics, e.g. communities ranged in terms of settlement type between urban (20%), peri-urban (30%) and rural (50%) settings^23^. As of June 2018 (when the data used for this analysis was downloaded from the system) the FRMT BL had been applied in 118 communities across 9 countries. Of those, 17 communities had conducted a PE due to a serious flood event and 68 communities had also completed an EL study. The very resource intensive task for setting up resilience measurement over different time periods on such detailed level hindered the inclusion of more datapoints for the PE and EL studies. However, our study is still the only one currently available that can analyze the dynamics of resilience in a globally consistent way. For example, the data generated from this endeavor already consists of more than 1.2 million responses on the household and community level, roughly 17,000 source grades and 442 graded resilient outcome variables. For the sake of comparison and consistency, the report focuses on those communities with completed BL and EL studies, and/or BL and PE studies. Thus, this paper focuses exclusively on the data collected from 68 communities who completed BL and EL studies, and the 17 communities who completed BL and PE studies. For static and multivariate statical analyses of resilience dimensions we refer to Campbell et al.^19^ and Laurien et al.^14^.

## Results

Due to the huge output produced during the analysis we frequently refer to the Supplementary for details and focus here on the main outcomes and give specific examples to provide clarity and to avoid confusion. As indicated, our approach focuses on 88 sources of resilience grouped into five capitals (see Keating et al.^18^). It should be noted that the results of community resilience measurement presented here were discussed with the experts on the ground to validate the outcomes as well as provide a narrative for the changes in resilience sources. That is, the NGO partners on the ground that are collecting the data in the communities have been working in those communities for a number of years. Thus, the measurements are not taking place in a vacuum but rather are part of a larger program to help build flood resilience in these communities.

### Changes between baseline and endline resilience levels

**Figure 2:**
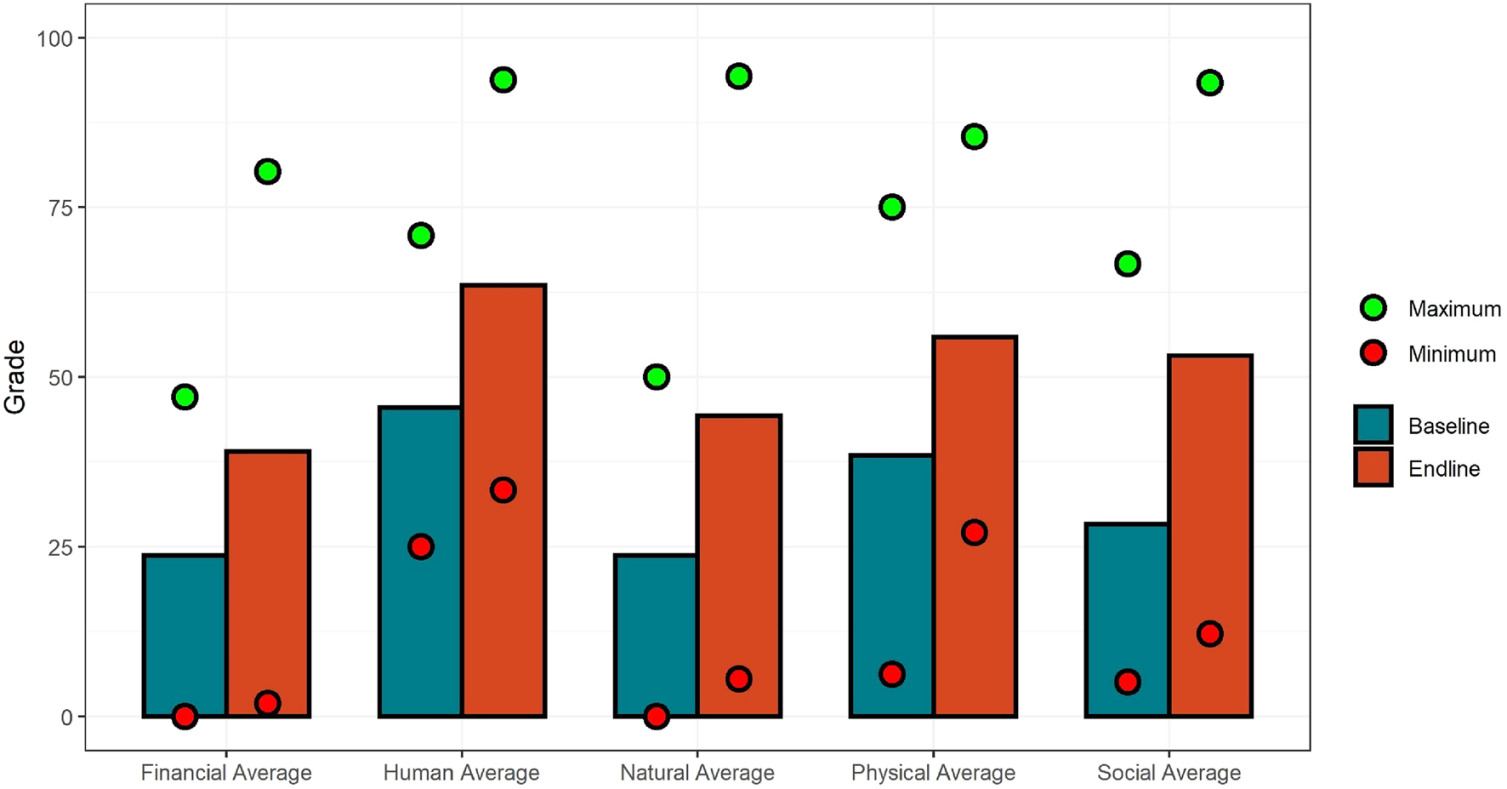
Comparing BL and EL average grades for the five capitals.

To start our discussion, Fig. 2 shows the average grades between baseline and endline studies grouped by the five capitals. Social capital saw the biggest absolute increase, on average, but no change in the median, pointing to the fact that a few community outliers are driving this change. Some of the changes can be also partly explained by learning curve effects (e.g. additional training on assessing natural capital sources was provided between the BL and EL data collections) as well as explicit actions taken to increase resilience between the BL and EL (e.g. social capital increases due to NGO actions to strengthen social connections).

Additionally, we also found increases in nearly all sources between BL and EL, irrespective of a flood disaster event occurring. However, the magnitude of increases is quite different across capitals and communities. Furthermore, the ranges (i.e. minimum and maximum mean grades in each capital) are quite different in the EL compared to the BL. In the BL the range between the maximum and minimum mean grades is smaller than in the EL, e.g. grades in the financial capital group in the BL have a minimum mean grade of 0 and a maximum mean grade of 48; in the EL data the range increases substantially from 0 to 80. The trend is similar for the other capitals (see Fig. 2).

In summary, we indeed find increases for aggregate mean grades, as well as for each of the five capital means and most of the individual sources of resilience between the BL and EL. Additionally, a larger spread in mean grades for each capital for the endline is found, indicating higher variation in the endline. This may suggest that all communities started out relatively similarly but differences in community development and flood risk management interventions, and increases in experience between the two periods, mean that grades diverged. Thus, while not conclusive, this provides some evidence of the efficacy of work being done to enhance flood resilience capacity as well as demonstrating the ability to benchmark and track flood resilience capacity in a consistent and systematic way.

### Baseline and post event outcome analysis

Related to the dynamics between the BL and EL is the question of how the sources may be linked to actual outcomes (impacts) when a flood event occurs (see Table 1). To explore this, we first looked at the correlations between the capitals and individual sources, as well as the overall grades of the PE. While we acknowledge that correlations do not yet indicate which sources are effective (causal) for flood resilience, it can demonstrate that some sources and outcomes move together in a systematic way (either positive or negative).

We summarize the findings from this analysis as follows. 75% of the physical capital sources are highly correlated with at least one resilient outcome; 69% of human capital sources are correlated; 65% of financial capital sources are correlated; 61% of social capital sources are correlated, and, 33% of natural capital sources are correlated with at least one resilient outcome variable. Interestingly, social capital, which has the greatest number of sources, has only one source that has four or more significantly high correlations with resilient outcomes (defined here as a correlation coefficient larger than 0.6 at the 5% confidence level). Perhaps not surprisingly (because of the link of losses and measurability of these indicators), financial capital has the most sources that have four or more high and significant correlations. The fact that there are sources from every capital that have significant and high correlations with outcome measures demonstrates the importance of measuring a holistic set of capacities for community flood resilience (for a summary we refer to Supplementary B). We also examined which resilient outcome indicators are most highly correlated with which sources. From this analysis, we find that many of the outcome measures show up as the most highly correlated variable with at least one source (see Supplementary C). This again points to the importance of measuring pre-event (sources) as well as post-event (outcome) characteristics to better understand how they contribute to actual resilience within a holistic perspective.

### Indications of non-linear dynamics

**Figure 3:**
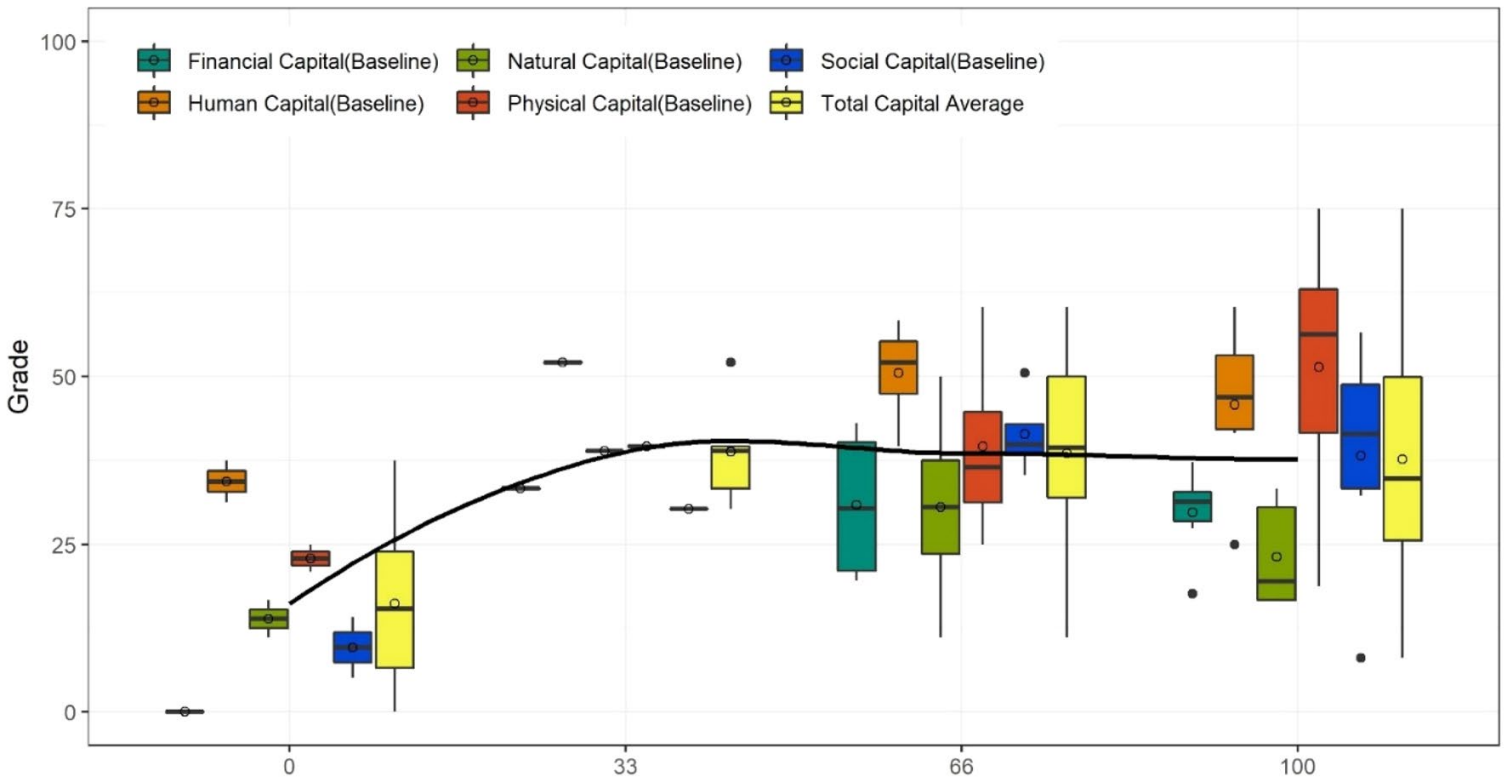
Relating the average capital grades with Death and Injury due to flood (O01).

Before moving to the advanced statistical analysis of our data we first indicate through a visual example, that there are strong indications that outcomes are related to the level of pre-event resilience in a non-linear way. While the correlations provide a good indication of what capitals are potentially more relevant for an outcome measure than others, the distribution of the capitals against outcomes gives a fuller picture. Visualization of non-linearity through boxplots was found to be especially revealing. As an example, Fig. 3 below shows the boxplots for each capital separated into the four grades (A-D, or 0, 0.33, 0.66 and 1) for one important resilient outcome measure—‘Death and injury due to flood’ (O01). By examining the median and the mean (the line and circle inside the boxes), it can be seen that the larger the resilience measured in the capitals, the larger a positive outcome observed (100 for the outcome measure means that the maximum possible resilient outcome was achieved—no or very minimal death and injuries). However, and as already indicated in the correlation analysis outlined above, financial capital in particular shows a good fit (see corresponding line) indicating that higher financial capital leads to lower death and injury tolls, however, in a non-linear way suggesting the interaction of other sources of resilience with financial capital is likely critical for achieving positive resilience outcomes.

As mentioned, we can only make indicative estimates given that there are only 17 PE studies. A backward stepwise regression analysis shows that financial capital is indeed the most significant variable for Death and injury due to flood (O01) but financial capital is by no means the most significant one for all outcome measures. For example, for Building losses and damage (O02) the most significant sources are physical capital, for Food security (O09) it is social capital. This indicates that post-event outcomes are also dependent in regards to the content-related capitals. However, we also found significant correlations of some outcomes to multiple capitals providing evidence that different types as well as combinations of content-related capitals are relevant. So far we have not included such multivariate and non-linear behavior in the analysis. We proceed now with an advanced statistical analysis using the data at hand to gather information about this possibility using all information available in the dataset.

### Dynamics from baseline to endline given post-events

Because we have communities that did and did not experienced a flood event, we have a quasi-controlled experiment with which to analyze the changes (called the dynamics) of BL and EL results and compare it to cohorts with and without flood events in the investigation period. Due to space restrictions we focus on financial capital in this section, as it is usually seen as one very important dimension of resilience (for the other capitals we refer to the Supplementary D). We start with some overall observations. Firstly, it is worth noting that the mean difference between the BL and EL for financial capital is higher in communities that have not experienced a flood event, approx. 14.6 (median 9.8, std. deviation 14.5); while in communities that have experienced a flood, the mean difference is approx. 13.3 (median 11.7, std. deviation 11.8). The situation is similar for the other capitals as well. In other words, compared to the no-flood event communities, the improvements in resilience grades for flood event communities were much smaller on average. This is an indication that the average increase in the sources is smaller in flood affected communities, providing support for our hypothesis that floods impact resilience capacities. However, using a series of parametric and non-parametric simple groups tests for the paired samples we found, that these differences are not statistically significant*.* One reason for this could be the large standard deviation and the small sample size which makes such testing procedures difficult to interpret. More data is needed here for future analysis.

Secondly, some increases in average grades can be explained by action taken between the BL and EL, as well as learning curve effects of graders. For example, feedback from partners explained that the social capital increases are likely due to the efforts of the NGOs engaging in the community, both directly via their community capacity building work, and indirectly through helping to strengthen the social channels through which they work. After social capital, the second biggest absolute increase was found in natural capital and is substantial across communities. Partners’ feedback indicated that it was difficult to find data for natural capital and thus to assess it in detail in the BL. Therefore, additional training on assessing natural capital took place in April 2017 prior to EL studies. Hence, the increase in natural capital grades is most likely largely attributable to this training and the results of the Boosted Regression Tree (below) supports this explanation. Natural capital was unique in that such comments and additional training were not observed for the other capitals and sources of resilience. Again, more data as well as further studies are needed to fully understand the multiple and complex relationships between sources of resilience and its measurement over time.

#### Resilience dynamics over time

To take these considerations into account, i.e. assuming in the case of learning curve effects they would be the same for all communities as well as that the change in ranges is the same across these communities, our starting point for the Boosted Regression Tree (BRT) analysis is the differences between the average capital scores of the BL and EL, where a positive/negative score means an increase/decrease in resilience capacity. These differences are considered as our dependent variables, while the BL capital scores, sources of resilience scores, the PE outcomes and socioeconomic variables are treated as our independent variables. In total there are 168 variables in the independent variable candidate set.

It is important to note before proceeding that using all the communities for the boosted regression tree approach did not result in very good predictive models. This indicates that different resilience indicators, e.g. depending on the geographical and socioeconomic context, may be relevant for the dynamics of resilience. From a conceptual perspective, this can be explained because communities are very heterogeneous and it would be unrealistic to assume that the same dynamics can be observed in all regions and socioeconomic facets. To investigate this matter in more detail, Laurien et al.^14^ developed a community typology of flood resilience using the baseline dataset and hypothesized that these typologies will also be important for the dynamics over time. We therefore followed the findings in Laurien et al.^14^ and separated the dataset into four community clusters based on resilience levels and socioeconomic characteristics (see Table 2). As we use a subset of communities used in Laurien et al.^14^ we checked that the clusters in our dataset are the same using also the Wald criterion.

**Table 2:** Typology of community flood resilience^14^.

Cluster name		# communities
Cluster 1	Very poor, struggling rural communities	29
Cluster 2	Poor but thriving rural communities	51
Cluster 3	Middle-income urban communities with less frequent flood risk	24
Cluster 4	Middle-income mixed type communities with more frequent flood risk	14

In a next step we linked the EL and PE data with the BL in these clusters to empirically test if and in what way different dynamics can be observed for each cluster, as suggested by Laurien et al.^14^. Using the community clusters in Table 2 we applied the BRT approach and the models’ performance increased significantly compared to the full dataset model. One interpretation of this result is that different dynamics for each cluster need to be assumed. This finding is important because it provides empirical support for the hypothesis that communities at different stages of development have different mixes of resilience capacity and therefore resilient capacity will be prioritized differently.

**Figure 4:**
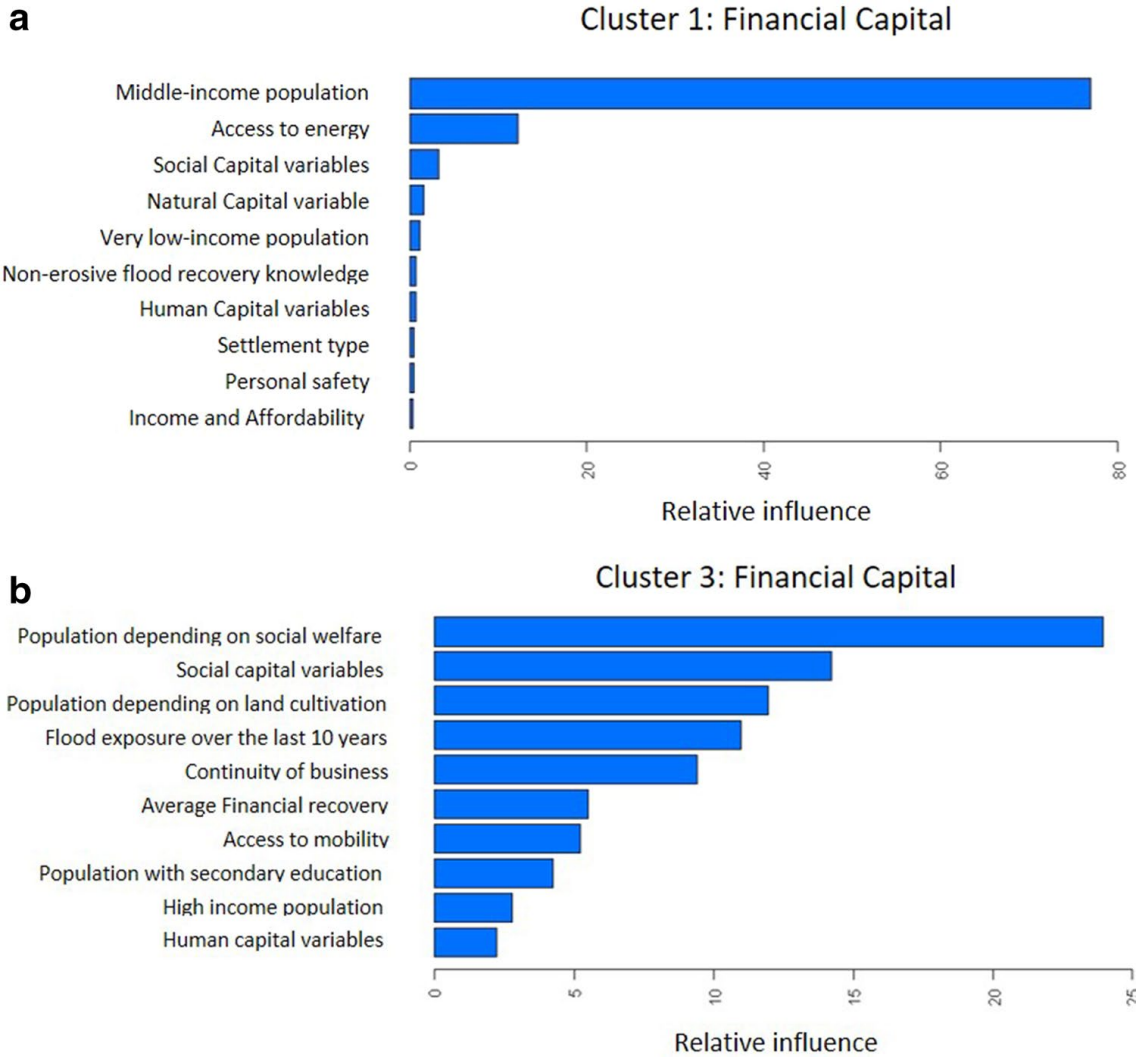
The 10 most important variables for cluster 1 and cluster 3 for the best fitted boosted regression tree model for financial capital.

Below we provide some examples using financial capital (the full analysis for all capitals and clusters can be found in Supplementary D and a summary is presented in Table 3 in the discussion section). Figure 4 (and the Figures in the Supplementary) shows the 10 most important sources of resilience in terms of influencing the Mean Squared Error of the given BRT model, i.e., the sources of resilience which showed the best predictive power for explaining the differences between the BL and EL.

As seen in Fig. 4, the most influential variables for cluster 1 (characterized by socioeconomic condition as very poor, struggling rural communities) are the relative proportion of middle-income population (by national standards) and the capacity of ‘appropriate and equitable access to energy’. By comparing it with the cluster description in Table 2 we see a dynamic interaction between poor people who strive for wellbeing-through higher incomes. Other sources such as financial ones have a very low influence which can be partly explained by the low capacity levels in all cluster 1 communities. In addition, cluster 1 has very little experience in past flood events and therefore it is not very surprising that outcome variables are less influenced. For cluster 3 (characterized by socioeconomic condition as middle-income urban communities with less frequent flood risk), we found more influential variables for explaining the changes in financial capital. For example, we found high explanatory power for the relative population living on social welfare, social capital in general, the proportion of people working on cultivating land, past flood experience, the continuity of businesses after an event, financial recovery time, community education rate and (appropriate and equitable) access to mobility. Again, we can find especially large dependencies of what drives financial capital. In cluster 3 which predominately consists of urban communities, socioeconomic characteristics (like public financial support) play an important role to foster financial stability during or after a flood disaster. It is worthwhile to note that no satisfactory BRT model for Cluster 4 was found, which partly can be explained by the small number of communities within this cluster and the possibility that for this cluster the dynamics actually do not follow an overall but rather individualistic pattern (as it also includes the wealthiest communities which have a diverse set of resilience options available). For the other cluster-capital dynamics and corresponding discussion we refer to the Supplementary Section D. A summary of the findings is presented in the Table 3 further down below.

#### Threshold effects in resilience levels

**Figure 5:**
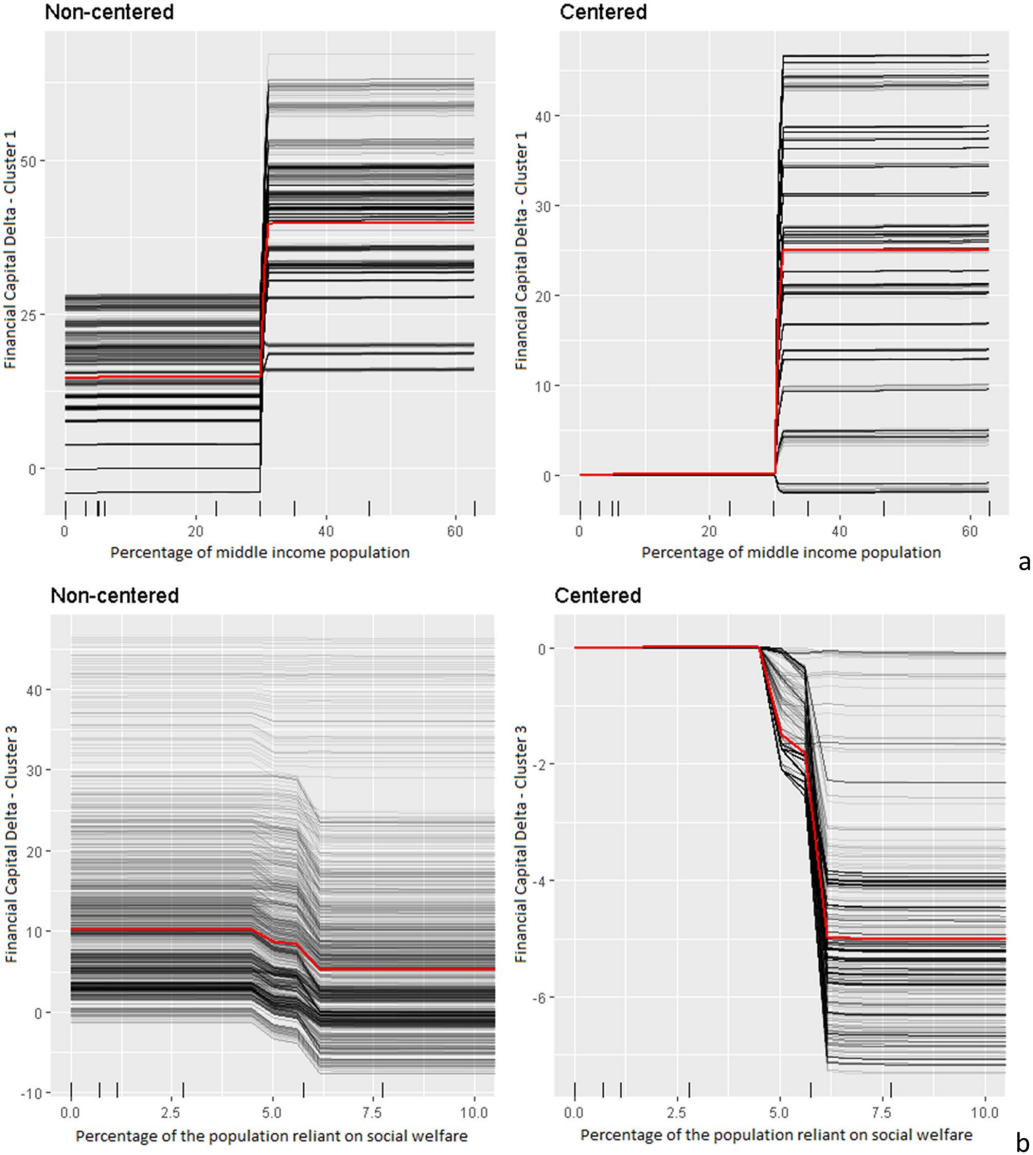
ICE curves for clusters 1 (**a**) and 3 (**b**) for the most influential variable of the best BRT model. The x axis shows the values of the independent variable and the y axis shows the predicted (expected) change while conditionally holding all other variables at their original values. The average of the ICE curves (red line) indicates the average predicted change for the variable value.

While there are strong indications that different dynamics of resilience need to be assumed for each cluster, a further important finding from our analysis is that the changes in resilience levels over time do not occur linearly but rather follow a ‘step’ pattern with specific thresholds. In other words, we found that based on the results of the BRT models, when a certain resilience capacity level or transformational threshold is reached, we can expect a triggering of increases in other capitals. To explain this process, we focus here on individual conditional expectation curves (ICE curves) from the BRT models. The ICE curve in Fig. 5 shows on the y axis how the prediction (expected value) changes as the variable on the x axis changes under the condition that all other variables remain fixed. The results are separated into non-centered ICE and centered ICE as the later can highlight heterogeneity in the results better, e.g. effects going into the opposite direction.

On the left hand side of Fig. 5a, we see that there is a distinct threshold value at 30%: when 30% of the population fall into the middle-income bracket, we see a large jump in the financial capital delta (on average 20 points). On the right hand side of Fig. 5a, we see that a few lines are showing a small jump into the opposite direction, however, most of them follow an increase of financial capital after this threshold is reached (i.e. the results are relatively robust). In Fig. 5b we see a very drastic drop in financial capital delta if 5% or more of the community population rely on social welfare as their primary source of income. These results as well as in Supplementary D indicate that specific sources of resilience or community characteristics are quite considerably related to an increase or decrease in capital levels.

## Discussion

Table 3 summarizes the overall findings focusing on the number of sources of resilience explaining (and indicating possible substitution effects) these dynamics the most (a threshold of 10% is selected here) and the most influencing source of resilience, (the detailed description of results can be found in Supplementary D).

**Table 3:** Summary of capitals and cluster related BRT results: each cell shows (i) the total number of sources found which had a significant predictive power (in square brackets) and (ii) the most important source of resilience within the best BRT model.

	Cluster 1 (poor, rural)	Cluster 2 (poor but thriving)	Cluster 3 (middle income, semi-urban)	Cluster 4 (rich, urban)
Financial capital	[2] Middle-income proportion of the population	[2] Average of all financial capital sources	[d] Percentage of the population living on social welfare	X
Natural capital	[2] Flood types	[1] Average of all natural capital sources	[3] Basin level flood controls	X
Social capital	[2] Proportion of very high income population	[1] Average of all social capital sources	[1] Financial recovery	X
Physical capital	[1] Educational attainment	[2] Middle-income proportion of the population	[4] Functioning and equitable education system	X
Human capital	[3] Proportion of very high income population	[2] Average of human capital sources	[3] Functioning and equitable water services	X

We find that in most cases not one but rather quite a few independent sources are important for different capitals and clusters. For cluster 1 a high diversity was found in terms of number and types of sources to be important. For cluster 2 the changes are very much related with the specific capital forms (e.g. a set of sources within specific capitals). Cluster 3 again shows quite some diversity and sources related to changes and are again source specific. As already indicated, for cluster 4 no satisfactory model could be found, indicating that the dynamics in this cluster are highly individualistic and specific, with no generalizable dynamics.

It should be noted that given the limited number of observations available and the inability of any statistical model to test causality, the specific sources of resilience which are found to be most important for each cluster can only be seen as indicative. Furthermore, the changes in resilience level were measured between a couple of years and long-term changes (e.g. after 3 years or even longer) were not looked at within this study design. Keeping these limitations in mind, we conclude that we found empirical indications that the overall dynamics of the sources of resilience are different and very much dependent on specific community characteristics and that there are threshold effects that show an increase in resilience rather dramatically once reached.

## Conclusion

We performed an analysis of the dynamics of resilience based on a standardized community flood resilience grading approach and corresponding measurement tool applied across the globe for two time periods including PE studies in case of a flood event. One of the major findings of our research is that building resilience capacity follows a dynamic structure that differs substantially for different types of communities based on their level of development. This has been theorized in the literature but was shown here empirically including how these dynamics differ. While data limitations preclude definitive results at this stage and therefore our results should be considered indicative, they identify new directions for understanding resilience, both from a conceptual as well as policy and implementation perspective.

Our results indicate that resilience dynamics are context specific, which has important implications regarding appropriate resilience-building initiatives. From a policy perspective, our analysis leads to recommendations regarding the prioritization of specific short- and long-term pathways as communities transition from one to another cluster type where different dynamics will be important. Our second major finding is that resilience does not change continuously or smoothly, but rather changes also occur abruptly when a given level of resilience reaches a specific threshold.

Regarding future work our results have important conceptual, policy and practical/implementation implications. We demonstrate that measuring resilience in a holistic way is important because there is no single source of resilience or even capital that is a silver bullet. Furthermore, depending on the level of development, sources of resilience take on different prioritizations. This means that it is important that policies and interventions are tailor-made rather than done in a broad-brush manner, as the most efficient increase in resilience through specific implementation of strategies is dependent on the underlying resilience levels and community characteristics^25^. Additionally, underlying dynamics may be more complex than previously anticipated and non-linearities including critical threshold levels^12^ may have to be emphasized more strongly to understand the actual real-world dynamics of resilience and realized risk. In this context it should be noted that our results and recommendations are based on an analysis of resilience measures 1 to 2 years after a flood event and there is the question if and how the results relate to long term resilience development. For example, in case of severe flood events much more time and resources are needed to recover. In this context we suggest to relate our resilience measurement framework explicitly to comprehensive risk management approaches that can take different risk realizations into account, either empirically or through advanced modelling approaches such as agent-based modelling^22^. Our paper has taken some first steps to contribute to this discussion but more research is needed to further deepen the understanding.

## Methodology

Our results for the BL and EL as well as the PE analyses are reported in a step by step manner. The analysis builds the case for a systematic approach for studying flood resilience capacity at the community level. First, we present an overall picture of the differences between the BL and EL grades and discuss the results from the PE data analysis. Secondly, we discuss how the BL data can provide indications of which sources of resilience are important for decreasing the severity of PE outcomes. In doing so, we use classic statistical approaches including correlation and linear regression models. Based on these results we thirdly construct different sub-groups and apply a boosted regression tree (BRT) approach for analyzing the combined effects of BE-PE-EL dynamics over time (see Fig. 2). This method was selected because it is a state-of-the-art approach for exploring possible non-linear effects over time that other approaches such as generalized linear models are unable to incorporate. Due to its importance for our study, we discuss the approach in more detail below and refer to the more classic statistical techniques used here to the many textbooks available.

Generally speaking, BRTs “can be understood as an additive regression model in which individual terms are simple trees fitted in a forward, stagewise fashion” (Elith et al.^26^, p.1). They have several advantages compared to classic techniques as they (1) can handle complex nonlinear relationships, (2) automatically consider interaction effect between the many variables, and (3) incorporate missing data. As the name indicates, the basic ingredients are regression trees that relate the dependent with the independent variables by including recursive binary splits, and boosting which is used to combine simple models by an adaptive method to improve predictive performance. While BRT approaches are complex, corresponding results can be clearly summarized to illustrate important insights. The predictive performance is also often superior to many traditional modelling methods (see Elith et al.^26^). Limitations of using such an approach can be related to the comparatively time-consuming and highly resource intensive task of running such models, and the somewhat arbitrary choice of split points which we circumvent by selecting a very fine scaled search algorithm to increase the robustness of the results.

When utilizing BTR methods, not only the best predictors but also split points are chosen for minimizing prediction errors. For example, one starts at the top of a tree (i.e. where all observations fall into a single region or dimension) and successively splits the predictors space into new branches. Importantly, for each step the best split at that particular region is chosen rather than the split based on the best split for all the next steps (hence it is sometimes called a ‘top-down, greedy’ approach). The criteria for selecting the split points for each potential variable can be based on different measures, most often the Mean Squared Error (MSE)—which we also selected for our analysis. Boosting means that the models, i.e., respective decision trees, are fitted iteratively to the training data. The main idea is that it is easier to find an average rough rule of thumb(s) rather than a single, highly accurate prediction rule. For example, if one introduces a function representing the loss in predictive performance, a so-called loss function, boosting can be seen as a numerical optimization technique for minimizing the loss function by adding, at each step, a new tree that best reduces the value of the loss function. This is done in a step by step way, i.e. the first regression tree with a given tree size maximally reduces the loss function and for each following step the focus is then on the residuals, or in other words the remaining variance in the response that is not yet explained by the model (similar to forward stepwise linear regression approaches).

In our case, the training data was set to be 85% of the total observations and the other 15% were therefore our out-of-sample testing set. We give some further information especially in terms of robustness in the sensitiviy analysis section and for an introduction to BRT refer to Ridgeway^27^, Myles et al.^28^ and the seminal work by Elith et al.^26^.

## Sensitivity analysis and limitations

To test the robustness of the BRT results a series of robustness checks were performed. Firstly, we used randomized in and out sampling to test the sensitivity of the results in regard to the training and prediction model. In other words, we trained 100 models each time randomly selecting (100 iterations of) the observations to be used for the training sample and each time conducted a grid search in order to find the optimal combination of hyper parameters corresponding to each iteration. We found that model performance remained constant, without large deviations of out of sample performance and that the grid search converged to similar hyperparameter combinations across the 100 models. Secondly, in order to prevent in sample overfitting, each model was trained using a fivefold cross validation approach, i.e. when evaluating each individual tree we use only a random 80% subsample of in-sample data, the remaining 20% are used to evaluate if the algorithm is starting to overfit. Similar to a “leave one out” approach if the algorithm starts to overfit to the “left out” data this indicates that one has reached the “end” condition. Thirdly, we run each cluster’s data with the BRT model found in the other clusters in order to check if the other cluster models performed equally well as the one found for the specific cluster. As a result, we can say that all results discussed above are significant. Hence, our results can be seen as quite robust at least in statistical terms.

Additionally, as mentioned above for each of the 100 models for each cluster we performed a separate grid search in order to find the optimal (best performing in terms of in sample MSE) hyperparameters corresponding to the specific in–out of sample iteration. These hyper parameters are Bagging, Shrinkage, Minimum observations and Interaction depth. The Bagging parameter controls the size of the subset of the variables each tree is allowed to use in its prediction and prevents overfitting. The Shrinkage parameters is the value of the boosting multiplier, it prevents overshooting in the loss function. The Minimum observations parameter dictates the minimum number of observations allowed to be split at the end of the tree node. The interaction depth dictates the maximum complexity each tree can have. The main findings from this computation is firstly that our models are robust and consistent, secondly that the data within clusters is homogenous and thirdly that models behind each cluster are different from that of another cluster.

However, still various limitations of our approach and analysis have to be acknowledged. Most important here is that while the number of communities as well as distribution over the world are unique, the total number of observations especially in regard to statistical analysis is small, specifically if the post-event studies are included. One of the drivers for the inclusion of the post-event studies in the analysis was indeed the hypothesis that if a flood event occurs in a community, ceteris paribus, the average difference in the resilience sources will be smaller or even negative than if no flood event occurred. This is based on the assumption that some resilience capacity is ‘used up’ in the flood event and thus lowers the level. An alternative hypothesis is that sources of resilience (resilient capacity) could actually be strengthened in a flood event. It was interesting to find that flood events play a rather minor role in regard to overall resilience level changes between baseline and endline. Again, the number of observations was small so the results are to be treated with caution. A specific look at some of the outcome measures as well as the BRT results nevertheless show that flood events rather decrease resilience levels. More data in the future should provide a clearer picture of these dynamics and corresponding sensitivities.

## Supplementary Information

Supplementary Information.
