# Supplementary material for: Differences in the dynamics of community disaster resilience across the globe
Source: Sci Rep. 2021 Sep 2;11:17625. doi: 10.1038/s41598-021-96763-0 (PMC8413441; doi:10.1038/s41598-021-96763-0)
Supplement: Supplementary file 1 — Supplementary Information. [file 41598_2021_96763_MOESM1_ESM.docx]

**Title:**

Differences in the Dynamics of Community Disaster Resilience across the Globe

**Authors:**

Stefan Hochrainer-Stigler (Corresponding Author)*

IIASA- International Institute for Applied Systems Analysis, Laxenburg, Austria

hochrain@iiasa.ac.at

Stefan Velev

IIASA- International Institute for Applied Systems Analysis, Laxenburg, Austria

velev@iiasa.ac.at

Laurien Finn

IIASA- International Institute for Applied Systems Analysis, Laxenburg, Austria

laurien@iiasa.ac.at

Karen Campbell

Wharton School, Risk Management and Decision Processes Center, USA

IHS Markit, USA

Karen.Campbell@ihsmarkit.com

Jeffrey Czajkowski

Wharton School, Risk Management and Decision Processes Center, USA

Center for Insurance Policy and Research, National Association of Insurance Commissioners, USA

jczajkowski@naic.org

Adriana Keating

IIASA- International Institute for Applied Systems Analysis, Laxenburg, Austria

keatinga@iiasa.ac.at

Reinhard Mechler

IIASA- International Institute for Applied Systems Analysis, Laxenburg, Austria

mechler@iiasa.ac.at

**Abstract:**

The consideration of disaster resilience as a multidimensional concept provides a viable and promising way forward for reducing risk and minimizing impacts today and in the future. What is missing is the understanding of the actual dynamics of resilience over time based on empirical evidence. This empirical understanding requires a consistent measure of resilience. To that end, a Technical Resilience Grading Standard for community flood resilience, was applied in a longitudinal study from 2016 to 2018 in 68 communities across the globe. We analyse the dynamics of disaster resilience using an advanced boosted regression tree modelling framework. The main outcome of our analysis is two-fold: first, we found empirical evidence that the dynamics of resilience build on a typology of communities and that different community clusters experience different dynamics; and second, the dynamics of resilience follows transitional behaviour rather than a linear or continuous process. These are empirical insights that can provide ways forward, theoretically as well as practically, in the understanding of resilience as well as in regard to effective policy guidance to enhance disaster resilience.

**Keywords:** Resilience, Dynamics, Empirical Analysis, Boosted Regression Tree, Flood Risk.

**Supplementary Section A: Selected Communities**

More than 350,000 households or approximately 1 million people are located in communities reached by the FRMC in phase I of the Alliance (the information here is based on Campbell *et al.*, 2019). The dataset includes more than 10,000 data points of source grades (88 grades in 118 communities) and around 1.25 million data points from the raw data collection.

**Table 1:** Summary of countries and communities where FRMC was applied, 2015-2018. Source: Campbell et al., 2019

| **Country** | **Number of  communities** | **Estimate of  total population (000’)** |
| --- | --- | --- |
| **Afghanistan** | 12 | 13 |
| **Bangladesh** | 9 | 39 |
| **Haiti** | 4 | 36 |
| **Indonesia** | 40 | 258 |
| **Mexico** | 19 | 7 |
| **Nepal** | 21 | 19 |
| **Peru** | 5 | 40 |
| **Timor-Leste** | 6 | 4 |
| **USA** | 2 | 640 |
| **Total** | **118** | **1 056** |

Table 1 summarizes the country and number of communities as well as estimated total population reached in the study (Source: Campbell et al. 2019). The majority of rural communities are in Afghanistan, Mexico, Nepal, Timor-Leste and Bangladesh, while the majority of urban communities are in Indonesia together with Peru, Haiti and USA. We refer to the discussion Laurien *et al.*, 2020 for more information on the community characteristics.

**References:**

Campbell, Karen A et al. 2019. “First Insights from the Flood Resilience Measurement Tool: A Large-Scale Community Flood Resilience Analysis.” *International Journal of Disaster Risk Reduction* 40: 101257.

Laurien, Finn et al. 2020 “A Typology for Community Flood Resilience.” *Regional Environmental Change 20.*

**Supplementary Section B: Social Capitals and most significant sources.**

To indicate which sources scored the highest in terms of significance and correlation we included below the nine sources with correlation coefficient larger than 0.9 and significance level of 0.01 percent. These are:

1. Social capital: ‘Mutual Assistance Systems and Safety Nets’ (S10),
2. Financial capital: ‘Community Development investment vehicles’ (F16) (which is the only source with over 80% correlation with two resilient outcome variables),
3. Physical capital: ‘Flood Emergency Infrastructure’ (P04),
4. Social capital: ‘Formal community emergency services integrate flood advice and management’ (S02),
5. Social capital: ‘Appropriate and equitable access to mobility’ (S12),
6. Social capital: ‘Functioning and equitable waste collection & disposal services’ (S18),
7. Human capital: ‘Personal Safety’ (H02),
8. Social capital: ‘Social Leadership’ (S24), and
9. Social capital: ‘Village and District Flood Plan’ (S26).

**Supplementary Section C:** Outcome measures and correlation to sources of resilience

We make the following observations and link specific sources of resilience to outcome measures:

- Financial capital sources are most highly correlated with 11 outcome measures. The financial sources of resilience were most often related to ‘Insurance’ (O26), followed closely by natural capital impacts, flood learning changes, death and injury and actions taken during the flood.
- Human capital sources are most highly correlated with 11 outcome measures. They most often related to natural capital losses, but also waste disposal, communication infrastructure, and insurance.
- With only 6 sources, natural capital sources are most highly correlated with five outcome measures. Interestingly, while only one natural capital source is associated with a natural capital outcome variable (O18), two are associated with the ‘Transportation and infrastructure loss and recovery variable’ (O11).
- Physical capital sources are most highly correlated with 9 outcome measures: ‘Waste disposal’ (O14) and ‘Educational provision’ (O06).
- Social capital sources are most highly correlated with 12 outcome measures and has proportionately the least number of outcome measures associated with it. Social capital sources are most often correlated with ‘Insurance’ (O26) followed by ‘Preparatory actions’ (O21). We can hypothesize about how many of the social capital sources may relate to the ability to organize and plan effectively for flood, however, non-linear aspects can be seen as equally important in that regard.

**Supplementary Section D:**

In this section the Boosted Regression Tree results according to different cluster types and capitals are discussed. We start with Cluster 1 and financial capital.

**Cluster 1 Results**

Very poor, struggling rural communities

**Cluster 1 (i) Financial Capital:**


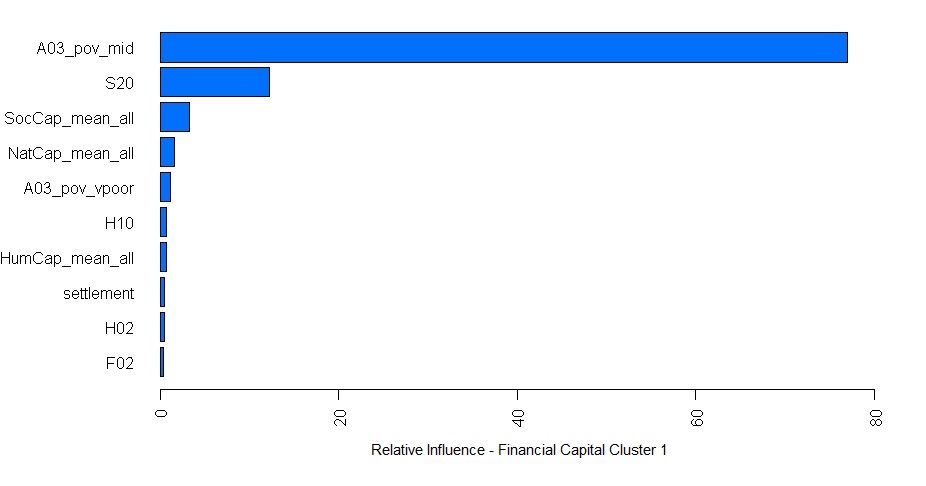


| **Variable Code** | **Variable Name** |
| --- | --- |
| A03_pov_mid | Percentage of middle-income population |
| S20 | Appropriate and equitable access to energy |
| SocCap_mean_all | Average of Social Capital Variables |
| NatCap_mean_all | Average of Natural Capital Variables |
| A03_pov_vpoor | Percentage of very low income population |
| H10 | Non-erosive flood recovery knowledge |
| HumCap_mean_all | Average of Human Capital Variables |
| settlement | Type of settlements: Urban, Peri-urban or Rural |
| H02 | Personal safety |
| F02 | Income and Affordability |

Note: The most informative variables are the middle-income proportion of the population and S20-Appropriate and equitable access to energy.


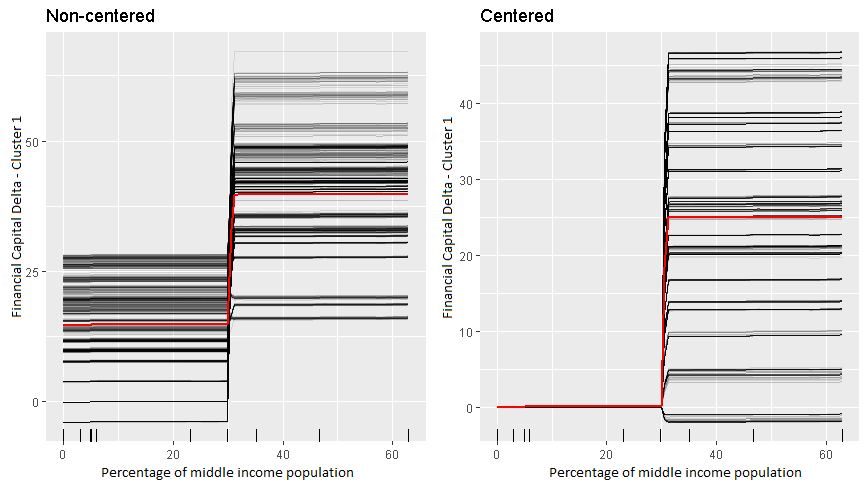


Note: A distinct break point at 30%: As soon as 30% of the population is in the middle-income group a large jump in the financial delta of around 20 points occur.


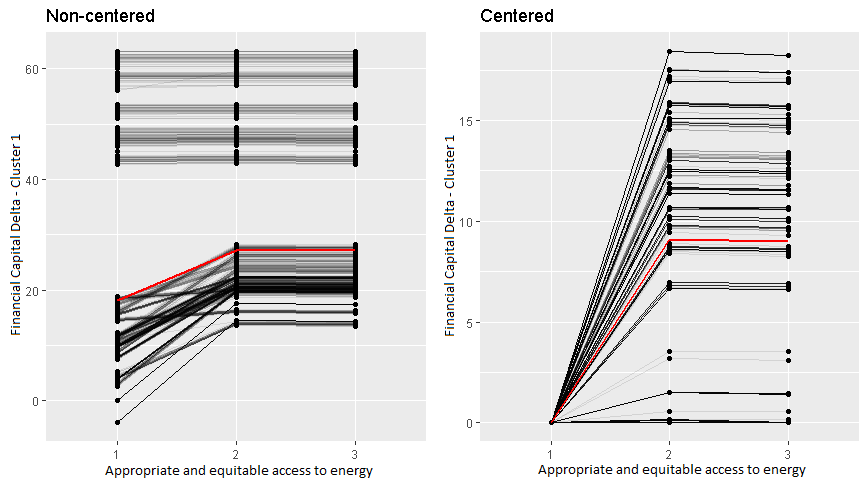


Note: The break point is for a community from a grading 1 (i.e. *A significant share of the community members have no appropriate access to energy*) to 2 (i.e. *Well functioning energy supply based on appropriate norms, standards and networks bring a satisfactory level of service to some, but are not fully equitable and not addressing the needs of all most vulnerable groups in the community*). The average increase in the delta is about 10 points. The communities in the upper part of the non-centered graph are outliers (e.g. those with an extraordinary increase for BL to EL)


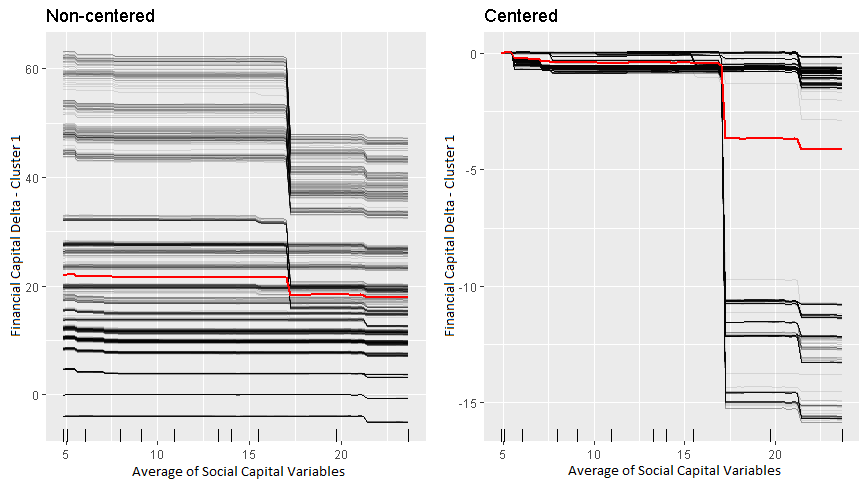


Note: The Financial Capital delta is negatively related with Social Capital. On the centered ICE graph a few communities from this cluster suffer a huge drop if the average social capital rises above 17, and all communities suffer a drop although smaller if the average social capital rises to 22.

**Cluster 1 (ii) Human Capital:**


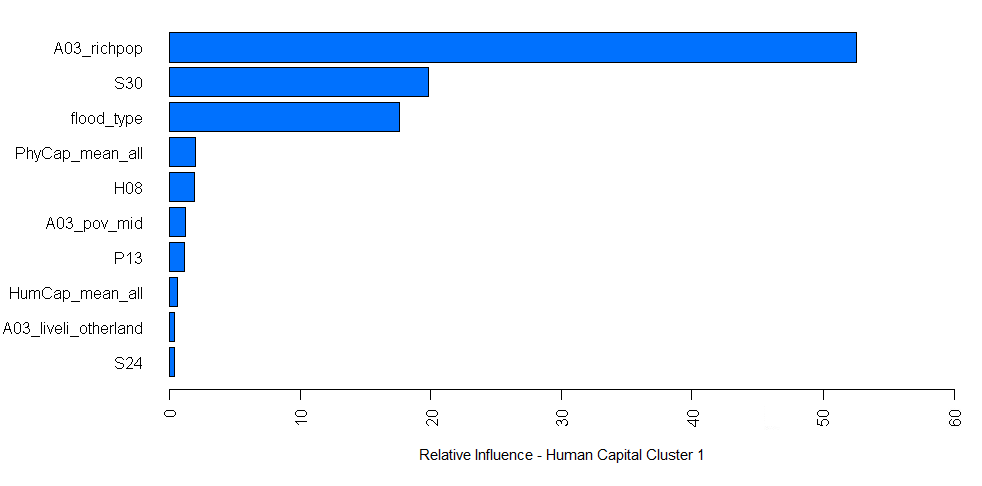


| **Variable Code** | **Variable Name** |
| --- | --- |
| A03_richpop | Percentage of very high income population |
| S30 | Government policies & planning and mainstreaming of flood risk |
| flood_type | What type of flood impacts the community: Flash flood, Pluvial flood or Fluvial flood |
| PhyCap_mean_all | Average of Physical Capital Variables |
| H08 | Flood vulnerability perception and management knowledge |
| A03_pov_mid | Percentage of middle income population |
| P13 | Water supply |
| HumCap_mean_all | Average of Human Capital variables |
| A03_liveli_otherland | Percentage of the population reliant on income from working on other people’s land |
| S24 | Social leadership |

Note: The most informative variables are the percentage of very high income (rich) population, “Government policies & planning and mainstreaming of flood risk” and the type of flood.


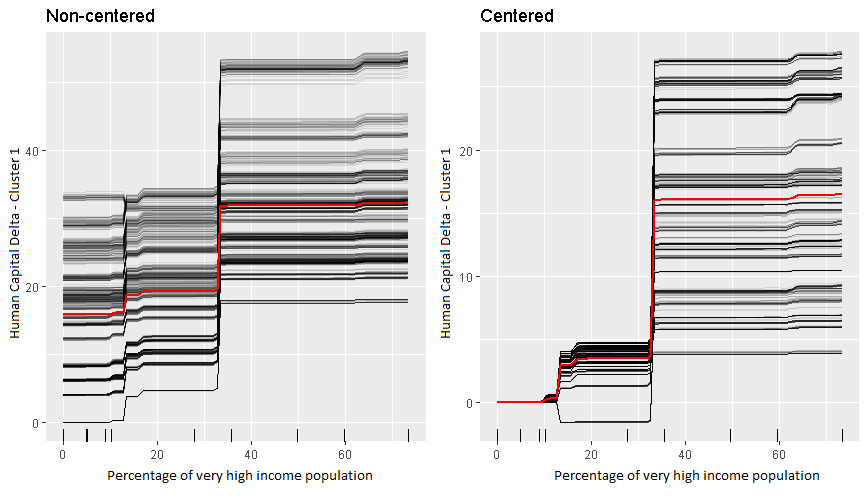


Note: Two distinct break points, a small one at 12% and another larger one at 37% can be found. As the community reaches 12% of the population being in the upper income group, there is a jump of an average of 5 points. Afterwards, as it reaches around 37% the human delta jumps by around 12 points.


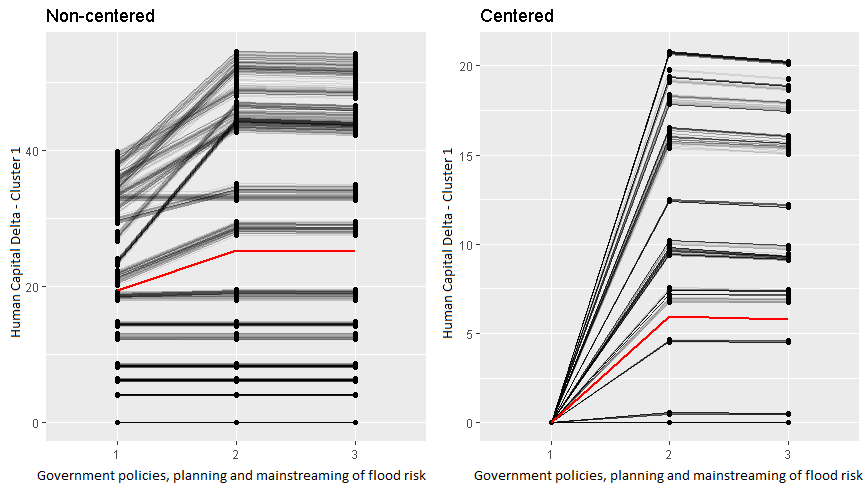


Note: A jump occurs from a grade 1 (or D in the questionnaire) (i.e. *Flood risks are not included in government policies and plans applicable to the considered community*) to a grade 2 (or C) (i.e. *Flood risk considerations are considered, but only in separate plans and policies, disconnected from other government policies*) of around 5 points, on average. However (left hand graph) the increase comes from communities which already have some capacity, i.e. an increase from BL to EL human capital is at least 20 (or 1 grade). For them, a jump of around 12 points occur. I should be noted that these communities are not the outliers which were found for financial capital.


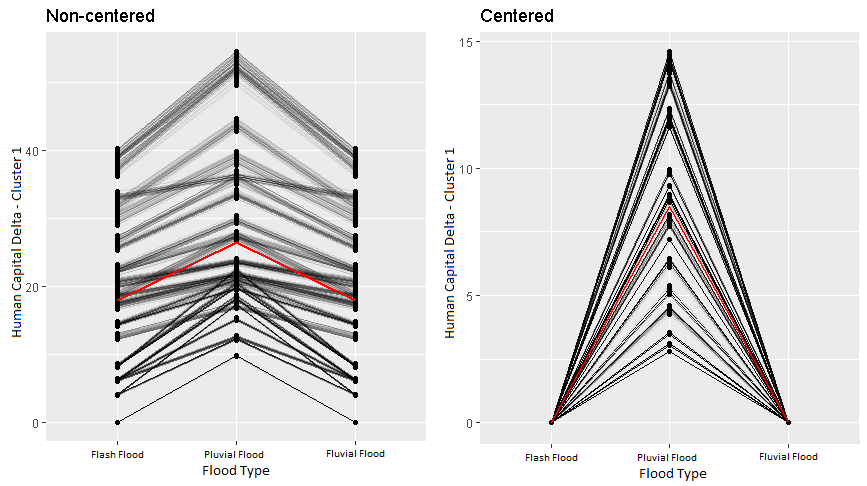


Note: The chart shows the flood type a community experiences: Flash floods, Pluvial (lake floods) of Fluvial flood (river floods).

**Cluster 1 (iii) Natural Capital:**


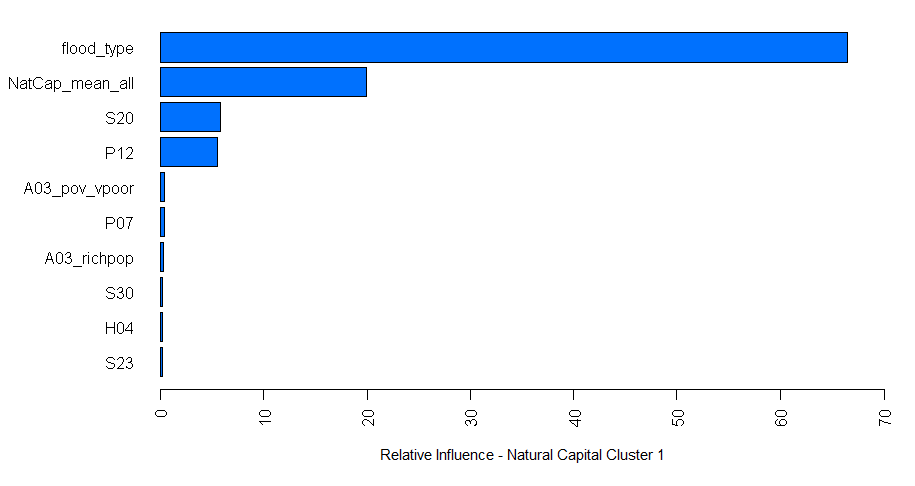


| **Variable Code** | **Variable Name** |
| --- | --- |
| flood_type | What type of flood impacts the community: Flash flood, Pluvial flood or Fluvial flood |
| NatCap_mean_all | Average of natural Capital Variables |
| S20 | Appropriate and equitable access to energy |
| P12 | Food security |
| A03_pov_vpoor | Percentage of very low income population |
| P07 | Communal Flood Protection (Flood controls) |
| A03_richpop | Percentage of very high income population |
| S30 | Government policies & planning and mainstreaming of flood risk |
| H04 | Value of education |
| S23 | Social inclusiveness |

Note: Variables with the highest influence on the natural capital delta are: the type of flood, the average of Natural capital at the Baseline followed by appropriate and equitable access to energy.


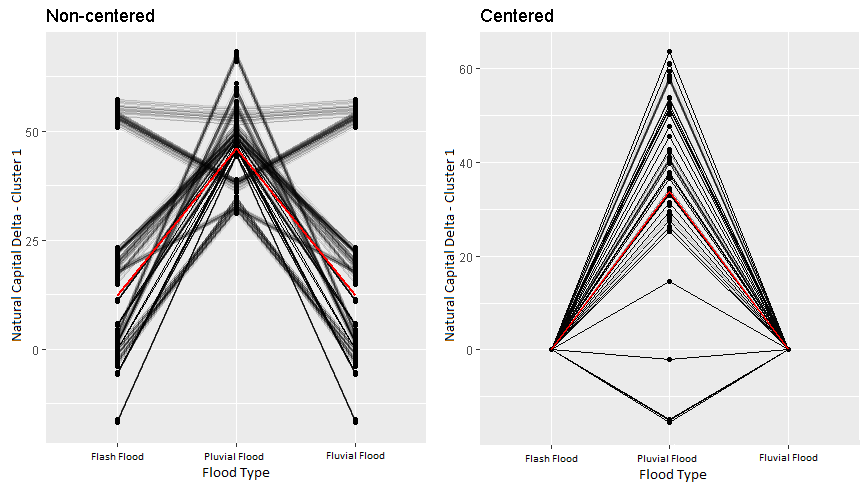


Note: On average Pluvial floods lead to the highest increase in Natural Capital, however, for some communities they are the most damaging ones and lead, on average, to a lower Natural capital delta compared to either flash or fluvial floods.


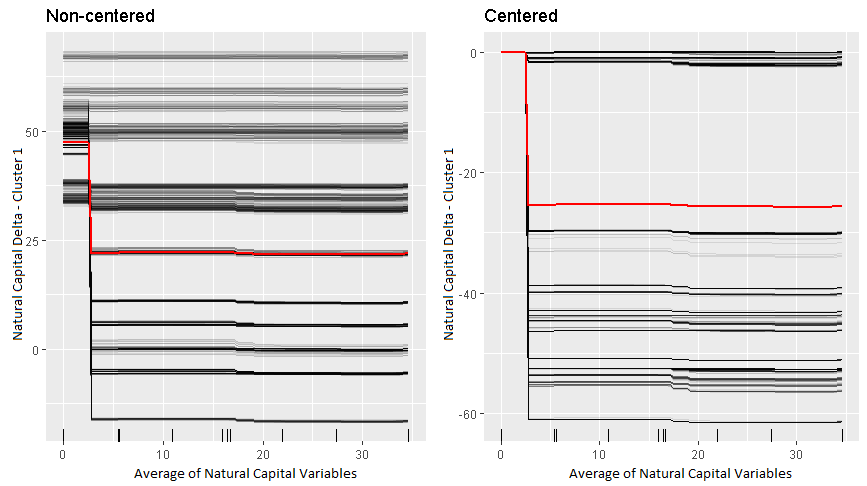


Note: The graph above shows the conditional expectation of the Natural Capital delta to changes in Natural Capital itself (i.e. at BL). One can hypothesize that there have been considerable efforts to increase Natural Capital given small scores at the BL.


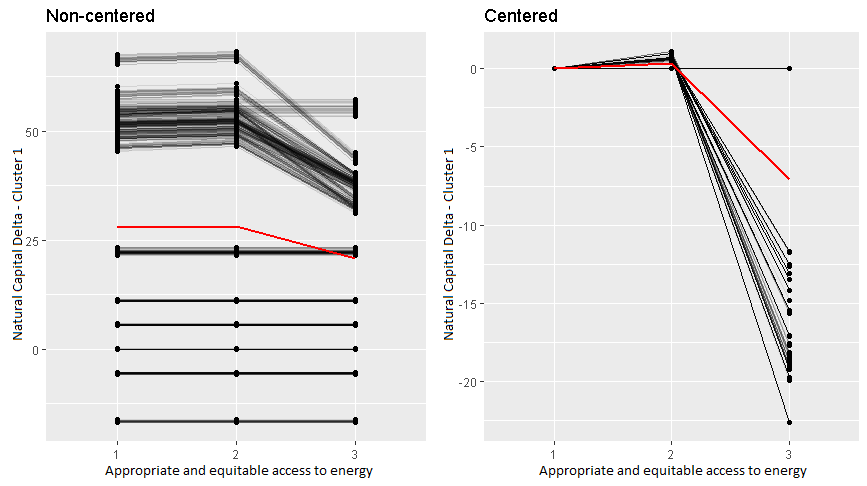


Note: Here we see that there is a drop when S20 (Appropriate and equitable access to energy) goes from 2 to a 3.

**Cluster 1 (iv) Physical Capital:**
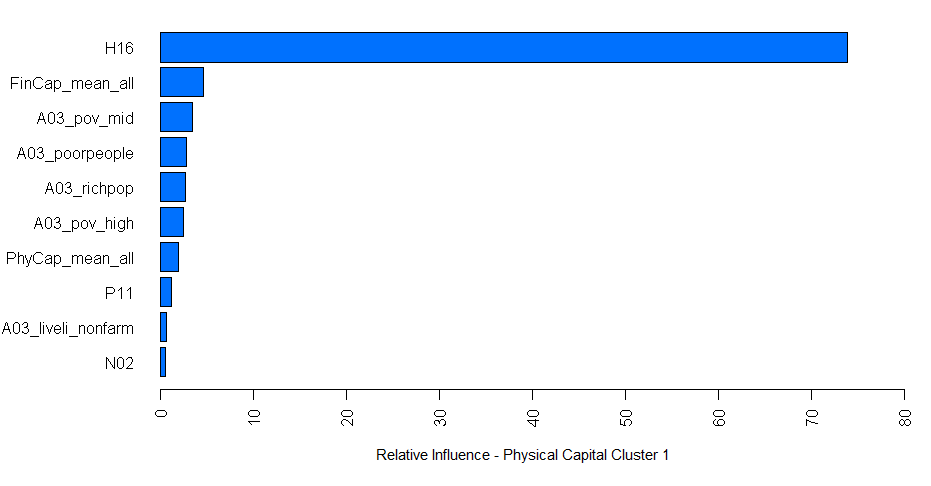


| **Variable Code** | **Variable Name** |
| --- | --- |
| H16 | Educational attainment |
| FinCap_mean_all | Average of Financial Capital Variables |
| A03_pov_mid | Percentage of middle income population |
| A03_poorpeople | Percentage of the community under the national poverty line |
| A03_richpop | Percentage of very high income population |
| A03_pov_high | Percentage of high income population |
| PhyCap_mean_all | Average of Physical Capital Variables |
| P11 | Lifelines infrastructure |
| A03_liveli_nonfarm | Percentage of the population reliant on income from non-farming work |
| N02 | Habitat connectivity |


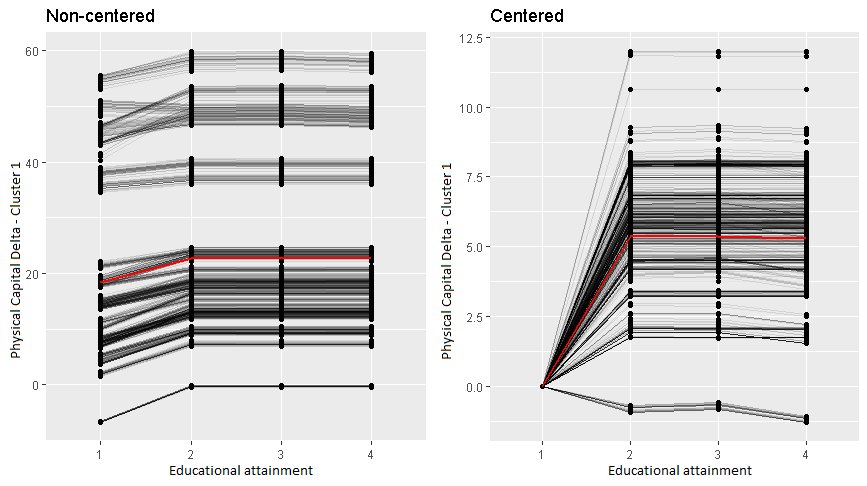


Note: Most informative variable for the growth of Physical Capital is H16 Educational Attainment. More specifically we see a jump when H16 goes from a D to a C.

**Cluster 1 (v) Social Capital:**


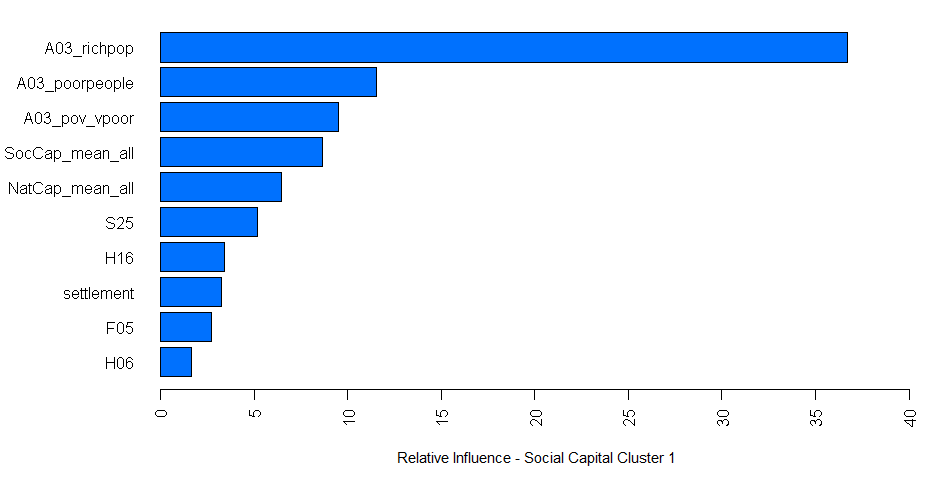


| **Variable Code** | **Variable Name** |
| --- | --- |
| A03_richpop | Percentage of very high income population |
| A03_poorpeople | Percentage of the community under the national poverty line |
| A03_pov_vpoor | Percentage of very poor population |
| SocCap_mean_all | Average of Social Capital Variables |
| NatCap_mean_all | Average of Natural Capital Variables |
| S25 | Culture for community information sharing |
| H16 | Educational attainment |
| settlement | Type of settlements: Urban, Peri-urban or Rural |
| F05 | Business credit access |
| H06 | Flood exposure perception |

Note: The most informative variables for Social Capital in Cluster 1 are the rich and poor populations.


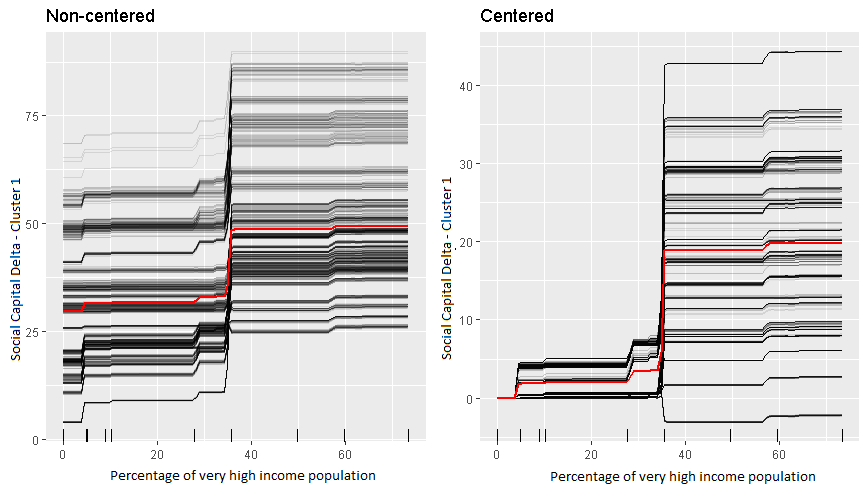


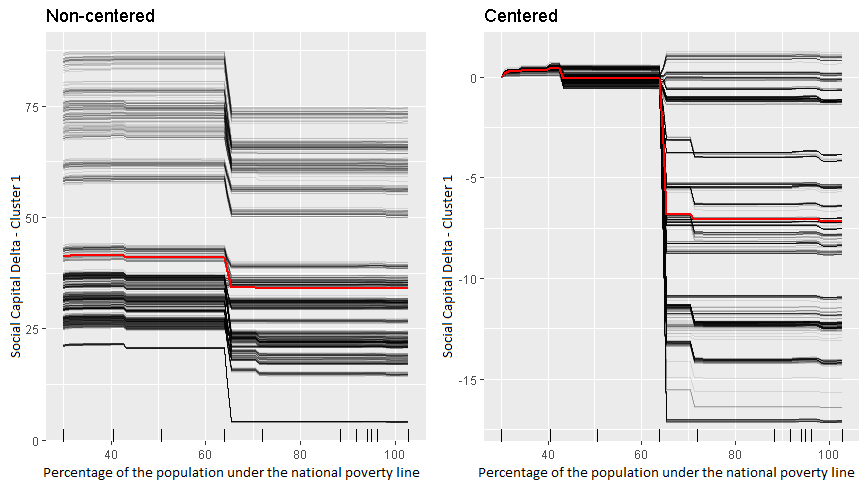


Note: Social Capital delta increases as the proportion of the very high income population grows and decreases as proportion of the population which is under the national poverty line grows.


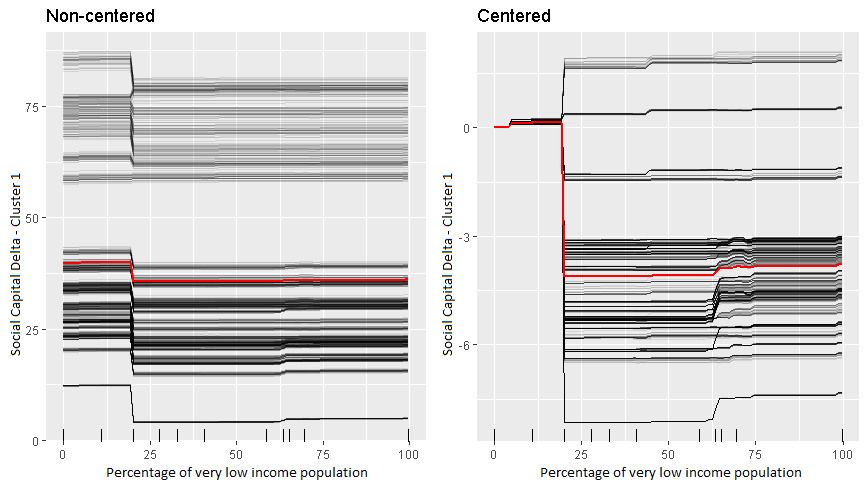


Note: The plot above shows the conditional expectation change of the Social Capital Delta in Cluster 1 with respect to change in the percentage of low income population.

**Cluster 2 Results**

Poor but thriving rural communities:

Cluster 2 is the most straightforward of the 4 clusters. The most informative variable for the delta of the capitals are the capitals at the Baseline themselves. Only selected plots presented here

**Cluster 2 (i) Financial Capital:**


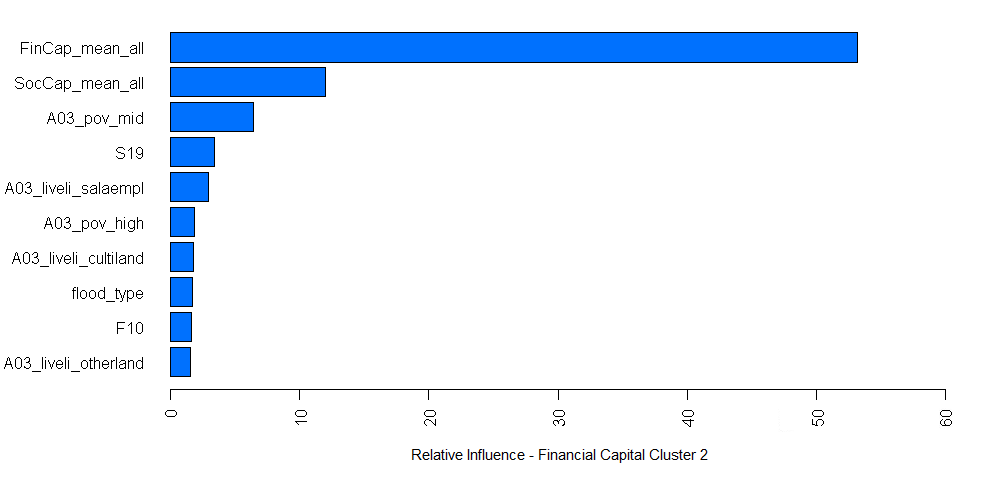


| **Variable Code** | **Variable Name** |
| --- | --- |
| FinCap_mean_all | Average of Financial Capital Variables |
| SocCap_mean_all | Average of Social Capital Variables |
| A03_pov_mid | Percentage of middle income population |
| S19 | Strategy to maintain or quickly resume local waste collection & disposal services in the event of a flood |
| A03_liveli_salaempl | Percentage of the population reliant on income from salaried employment |
| A03_pov_high | Percentage of high income population |
| A03_liveli_cultiland | Percentage of the population reliant on income from cultivating their own land |
| flood_type | What type of flood impacts the community: Flash flood, Pluvial flood or Fluvial flood |
| F10 | Continuity of business |
| A03_liveli_otherland | Percentage of the population reliant on income from cultivating other people’s land |


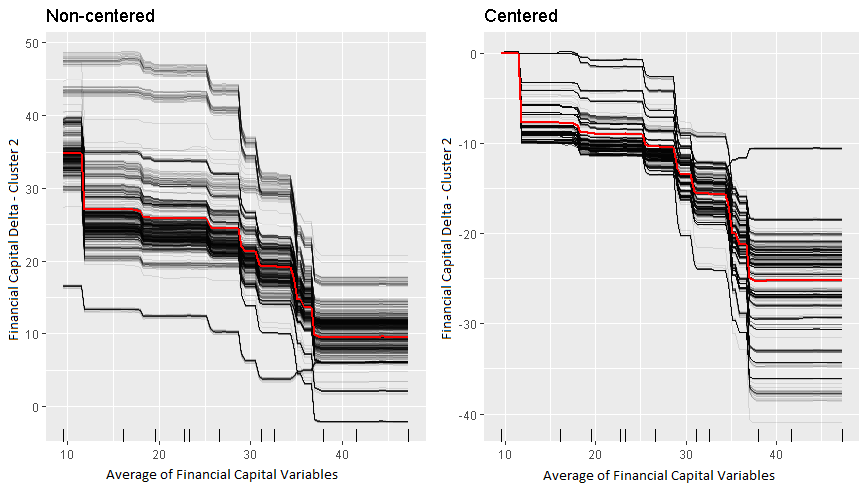


Note: Communities with lower initial values grew more.


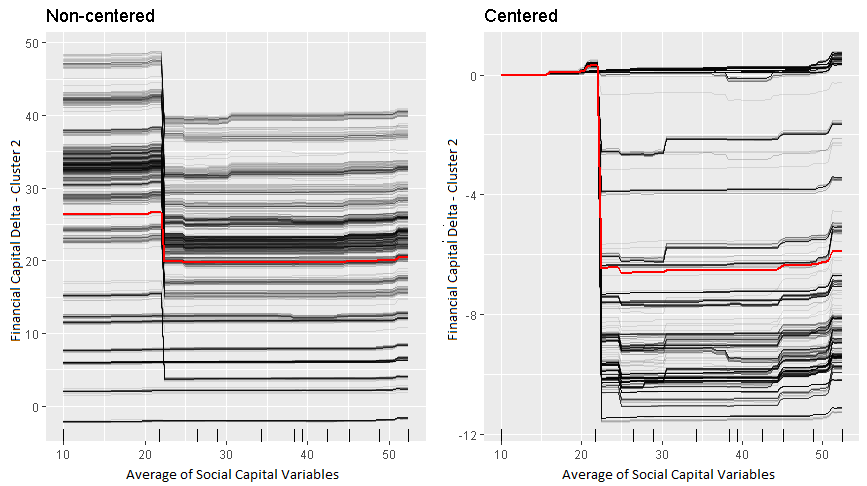


Note: A similar relation between Financial capital delta and the Social capital average as in Cluster 1: a drop at ~22, however, the drop is larger in magnitude (on average ~-6), whereas before the magnitude was on average ~-1.2


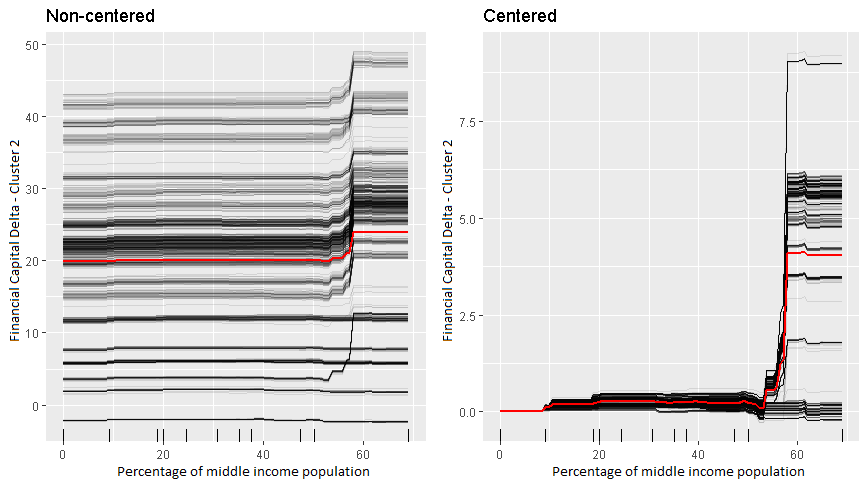


Note: If just above 50% of the population are in the middle income then we see a Financial Capital delta which would be on average ~4 points larger.

**Cluster 2 (ii) Human Capital:**


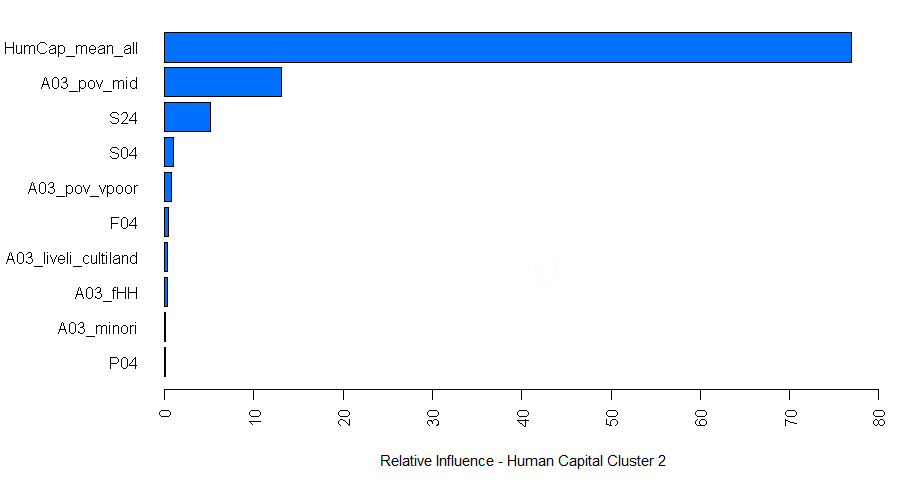


| **Variable Code** | **Variable Name** |
| --- | --- |
| HumCap_mean_all | Average of Human Capital variables |
| A03_pov_mid | Percentage of middle income population |
| S24 | Social leadership |
| S04 | Strategies for the delivery of actionable information for flood management |
| A03_pov_vpoor | Percentage of very low income population |
| F04 | Household Credit Access |
| A03_liveli_cultiland | Percentage of the population reliant on income from cultivating their own land |
| A03_fHH | Percentage of female headed households in the community |
| A03_minori | Percentage of the population considered minorities |
| P04 | Flood Emergency Infrastructure |


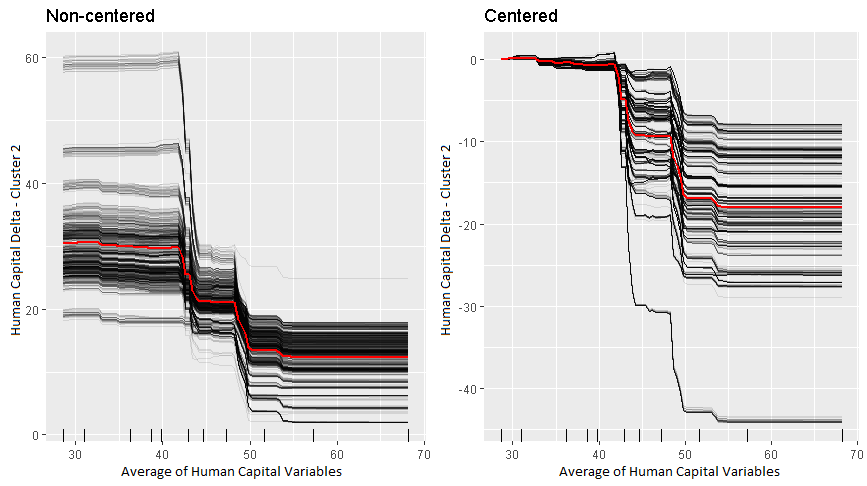


Note: Again the most influential variable for the Human capital delta is the Human Capital Average of the Baseline.


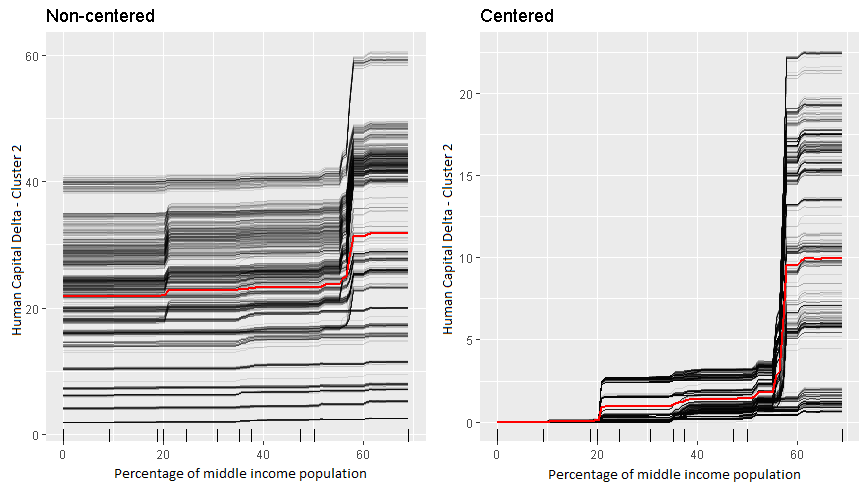


Note: Again, a large increase when the percentage of middle-income population reaches 50%, similar to the way Financial Capital behaves. Unlike the Financial Capital Delta, however,r here one also sees a sharp jump at 20%.

**Cluster 2 (iii) Natural Capital:**


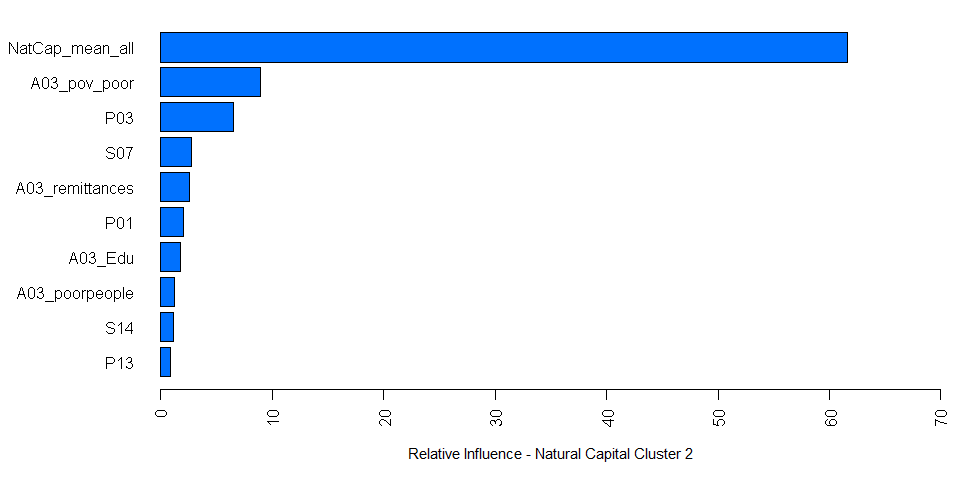


| **Variable Code** | **Variable Name** |
| --- | --- |
| NatCap_mean_all | Average of Natural Capital Variables |
| A03_pov_poor | Percentage of low income population |
| P03 | Measurement & Forecasting |
| S07 | Strategy to maintain or quickly resume healthcare services interrupted by flooding |
| A03_remittances | Percentage of the population reliant on income from remittances |
| P01 | Access to healthcare facilities |
| A03_Edu | Percentage of the population which have completed Secondary Education |
| A03_poorpeople | Percentage of the community under the national poverty line |
| S14 | Functioning and equitable food supply systems |
| P13 | Water supply |


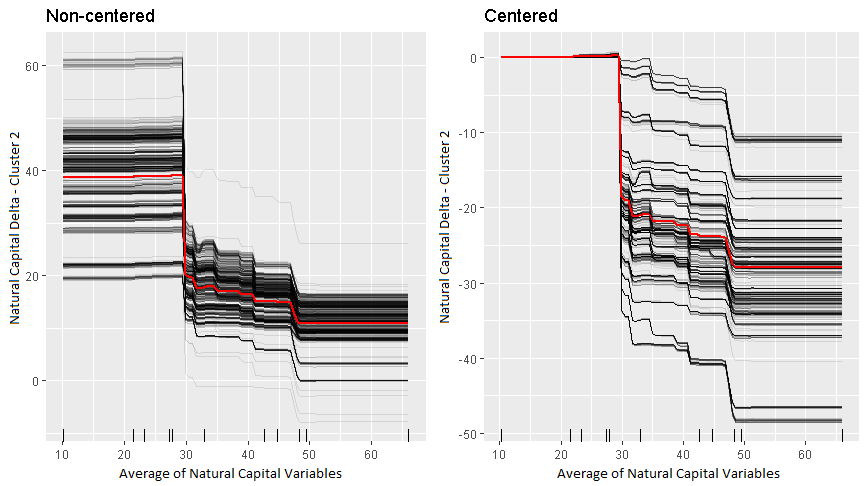


Note: Most influential variable for the Natural capital delta is the Natural Capital Average of the Baseline.


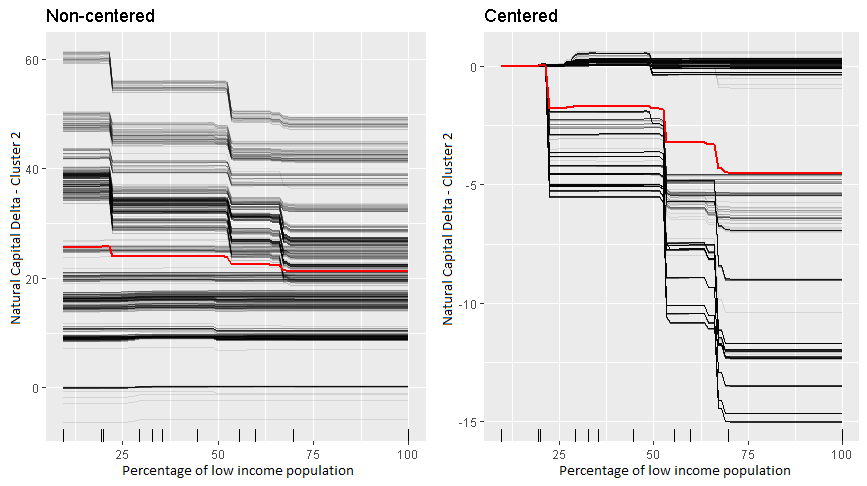


Note: One can clearly see several sudden decreases with drops at ~19%,~52% and 63%

**Cluster 2 (iv) Physical Capital**


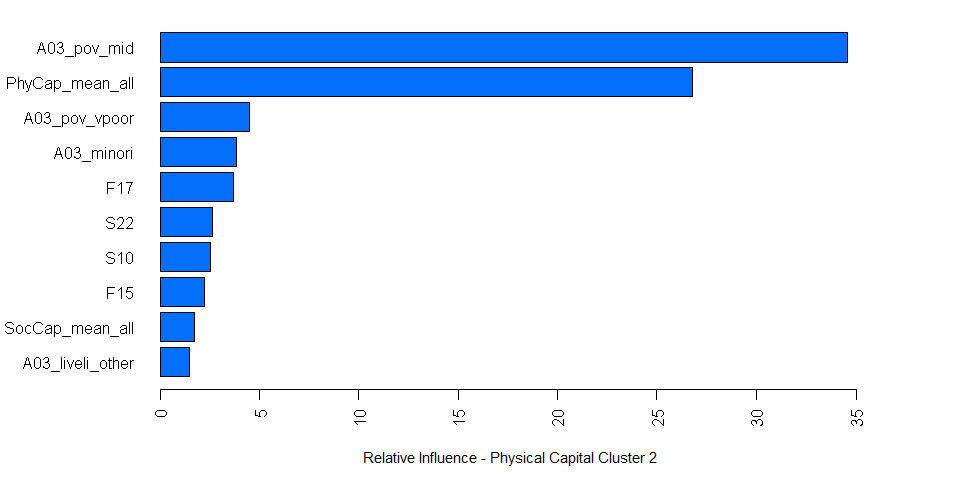


| **Variable Code** | **Variable Name** |
| --- | --- |
| A03_pov_mid | Percentage of middle income population |
| PhyCap_mean_all | Average of Physical Capital Variables |
| A03_pov_vpoor | Percentage of very low income population |
| A03_minori | Percentage of the population considered minorities |
| F17 | Conservation budget |
| S22 | Community representative bodies/structures for flood management coordination |
| S10 | Mutual assistance systems and safety nets |
| F15 | Government appropriations for infrastructure maintenance |
| SocCap_mean_all | Average of Social Capital Variables |
| A03_liveli_other | Percentage of the population reliant on income from other sources (that is not one of the following sources:   - Salaried employment (government, private business, NGO) - Cultivate own land - Work for daily wages on other people's land - Work for daily wages in non-farm work - Self-employment (i.e. artisan, small shop) - Savings/retired - Government or charity welfare/social security) |

Note: Most informative variables for the delta of physical capital are the middle-income population bracket and the state of Physical capital during the Baseline.


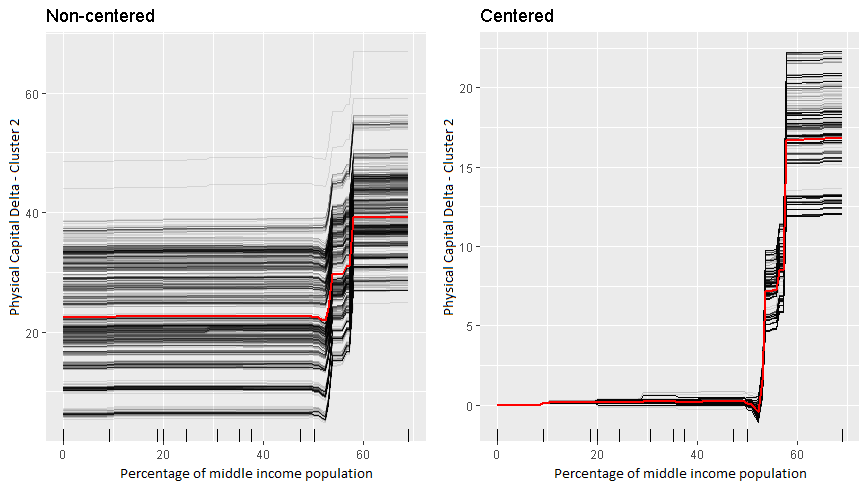


Note: A clear and sizeable jump in the physical capital delta as the community reaches ~54% of the population being in the middle income. One interpretation could be that in order for physical capital to grow one needs a minimum wealth requirement which could be gathered from the population (for example by taxes) such that it could be invested in infrastructure.


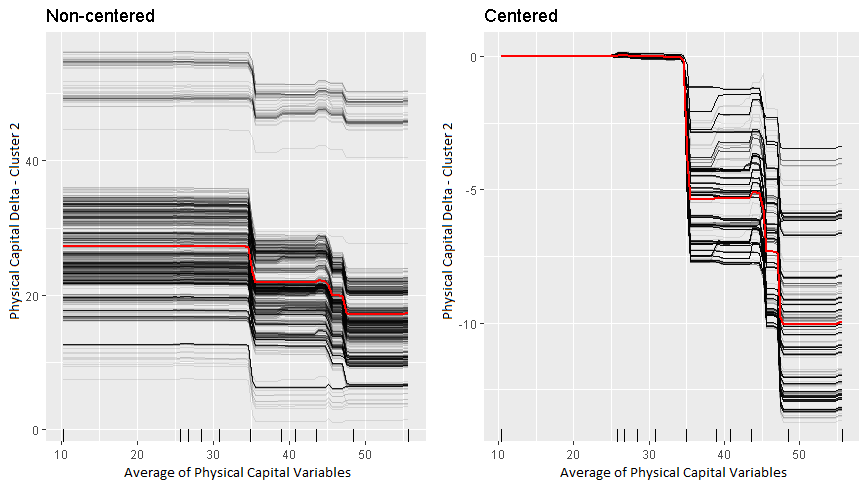


Note: Again, the most influential variable for the Physical capital delta is the Physical Capital Average of the Baseline.


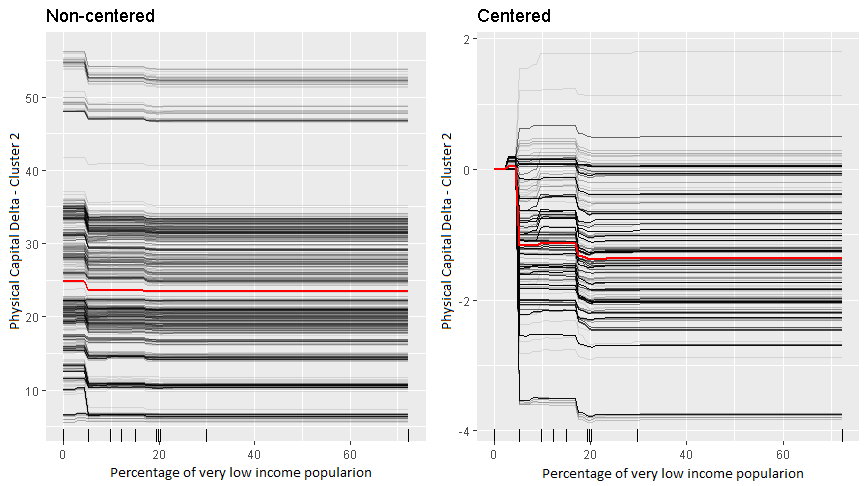


Note: Relation between the Physical Capital Delta and the percentage of low-income population. This variable is influencing the Capital delta only when it takes values from 0% to 20%.


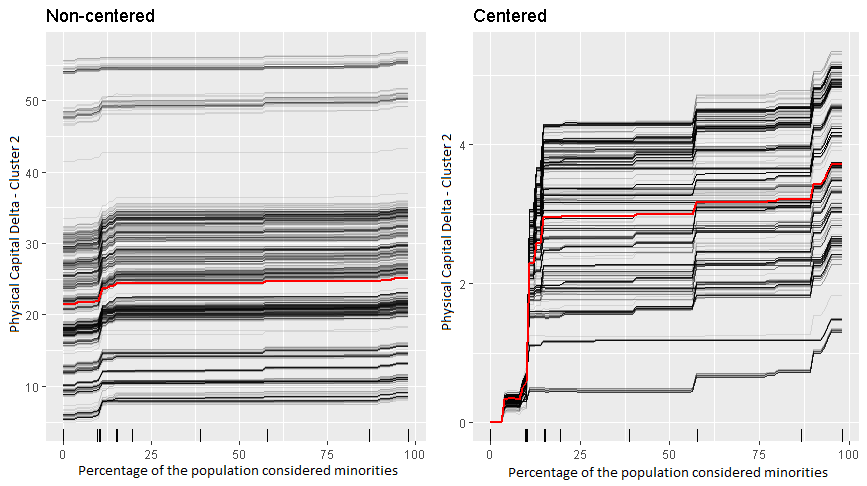


Note: This graph shows the change in Physical delta as the minority population increases. It appears that if communities have ~10% population of minorities their physical delta jumps by about 3-4 points.

**Cluster 2 (v) Social Capital:**


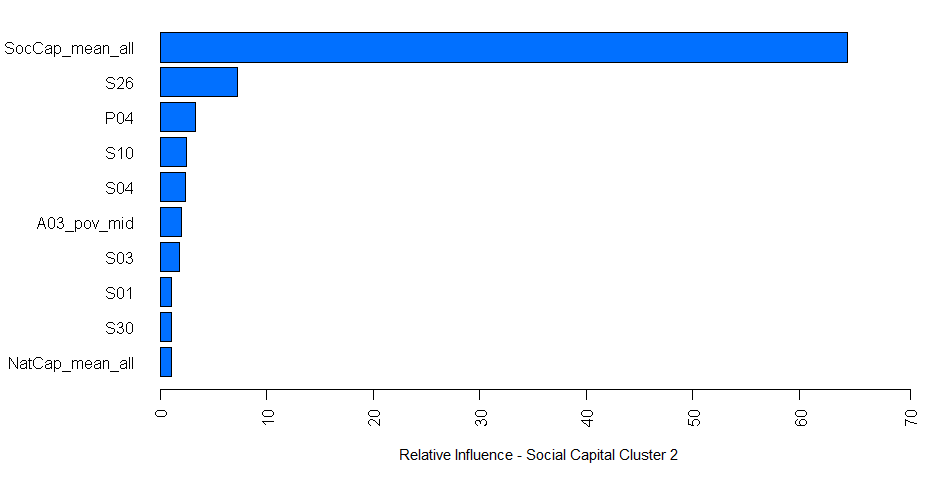


| **Variable Code** | **Variable Name** |
| --- | --- |
| SocCap_mean_all | Average of Social Capital Variables |
| S26 | Village or District Flood Plan |
| P04 | Flood Emergency Infrastructure |
| S10 | Mutual assistance systems and safety nets |
| S04 | Strategies for the delivery of actionable information for flood management |
| A03_pov_mid | Percentage of middle income population |
| S03 | Access to external, formal flood related services |
| S01 | Social participation in flood management related activities |
| S30 | Government policies & planning and mainstreaming of flood risk |
| NatCap_mean_all | Average of Natural Capital Variables |


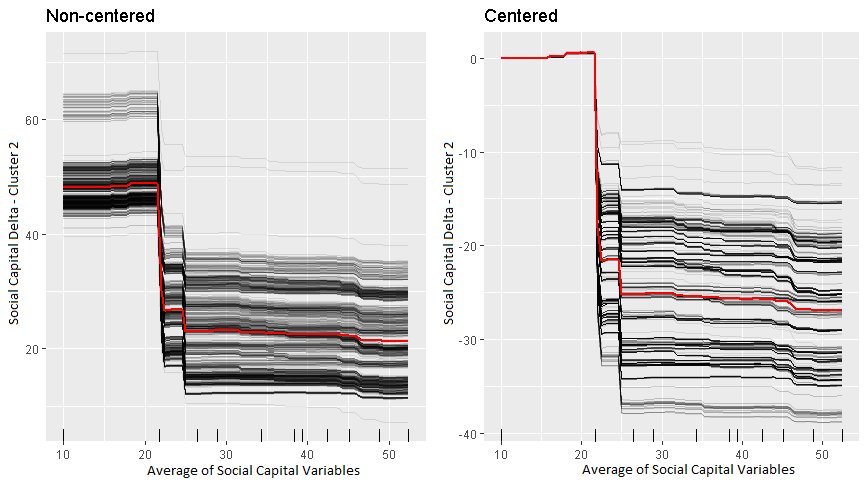


Note: The most influential variable for the Social capital delta is the Social Capital Average of the Baseline.


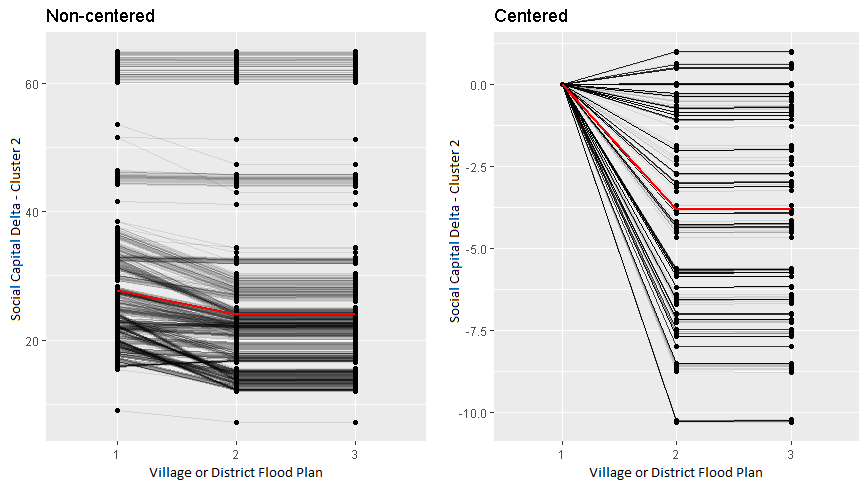


Note: Here we see a negative relation between the Social Capital Delta and S26-Village or District flood plan. The largest change comes when the variable goes from 1(D) to 2(C) that is from “No Village or District Flood Plan in place” to “Village or District Flood Plan is in place and fairly accepted, but its development has not been participatory and inclusive enough, and the plan has not been communicated and socialized widely within the community.”

**Cluster 3 Results**

Middle income, peri-urban and urban communities with less frequent flood risk

**Cluster 3 (i) Financial Capital:**


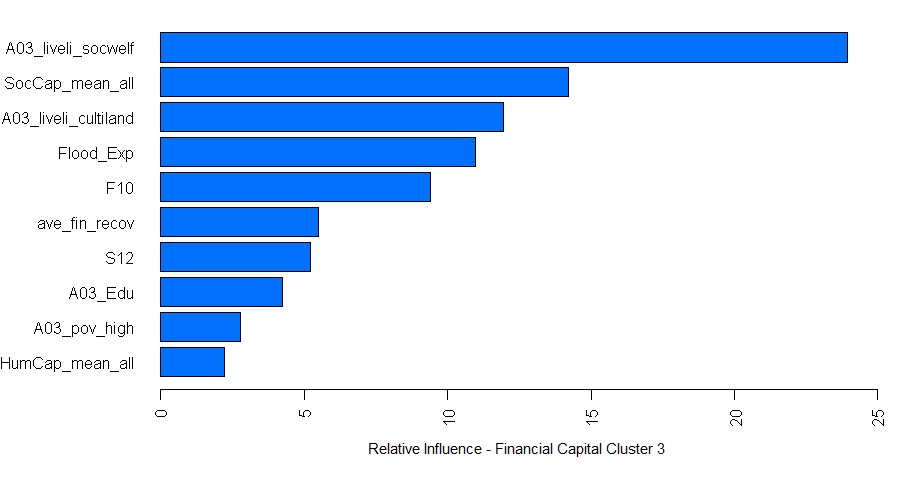


| Variable Code | Variable Name |
| --- | --- |
| A03_liveli_socwelf | Percentage of the population reliant on social welfare |
| SocCap_mean_all | Average of Social Capital Variables |
| A03_liveli_cultiland | Percentage of the population reliant on income from cultivating their own land |
| Flood_Exp | Flood Exposure |
| F10 | Continuity of business |
| ave_fin_recov | Average financial recovery |
| S12 | Appropriate and equitable access to mobility |
| A03_Edu | Percentage of the population with completed secondary education |
| A03_pov_high | Percentage of high income population |
| HumCap_mean_all | Average of Human Capital Variables |

Note: The most informative variables in Financial Capital in Cluster 3 are: Percentage of the population living on social welfare, The mean of the social capital, The proportion of people living cultivating land, Flood exposure, F10 (Continuity of business), average Financial Recovery, Education and S12 (Appropriate and equitable access to mobility)


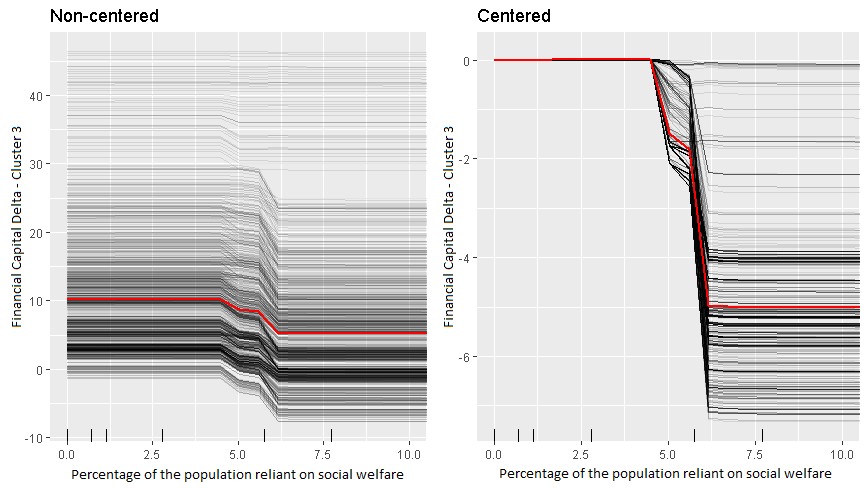


Note: A very drastic drop in Financial Capital delta if in the community 5% or more of the population rely on social welfare as their primary source of income.


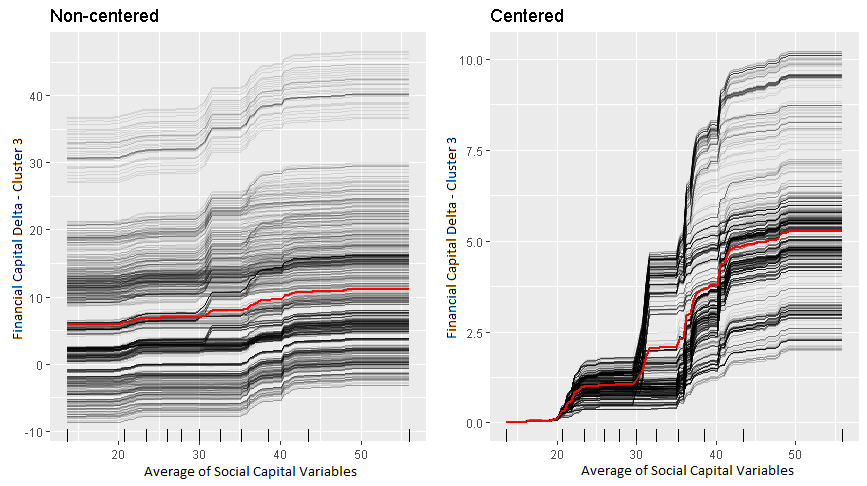


Note: Strong positive effect between the Average Social Capital during the baseline and the Financial Capital Delta. We can see jumps at 20, 30,35 and 40 points.


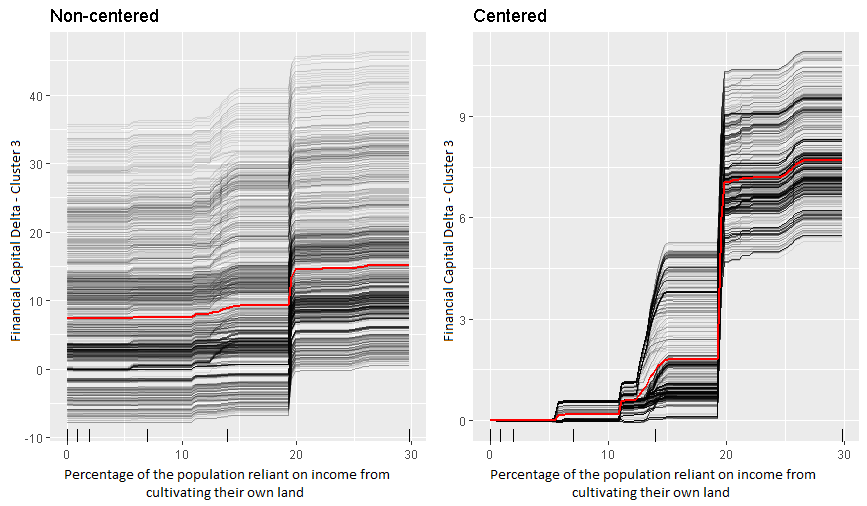


Note: Positive jump in the Financial Capital delta as about 18% of the community’s primary source of income is cultivating land.


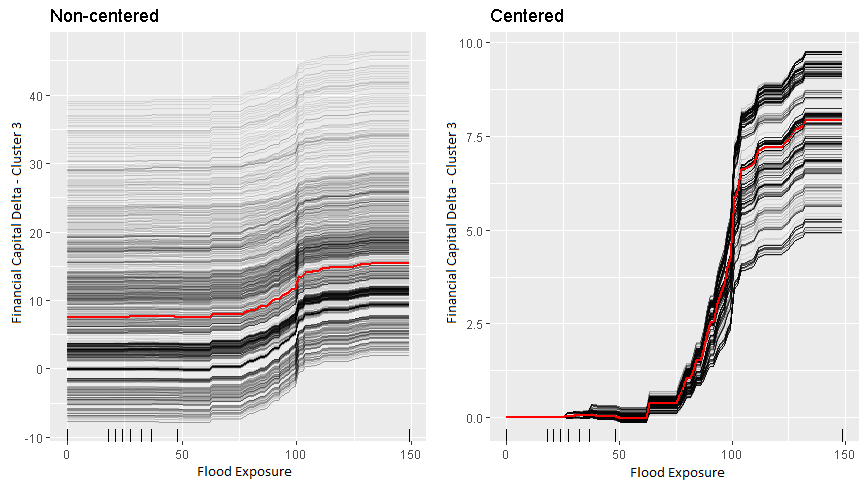


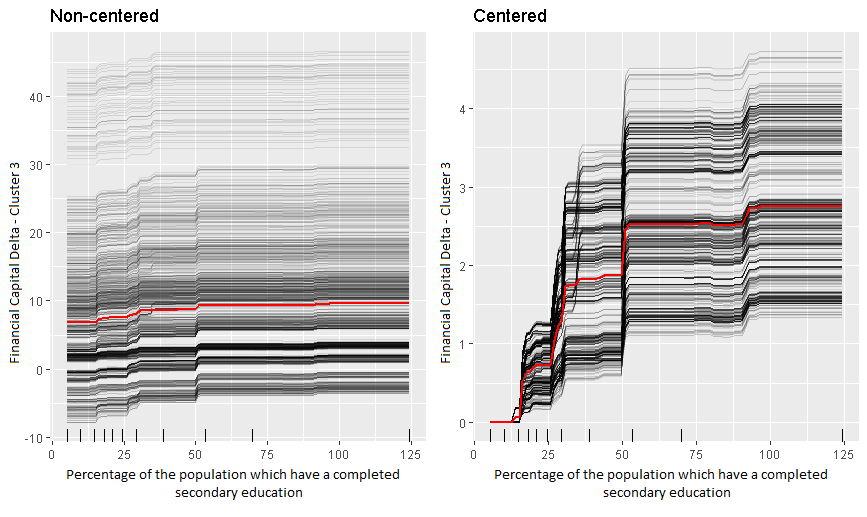


Note: Positive effect in financial delta if the education levels increases in the community. There are jumps at 12%, 25%, 30%, 50% and 93%. Note that the graph was extended to 125% for better visualization. There is no effect beyond 100% hence the lines are straight.

**Cluster 3 Results (ii) Human Capital:**


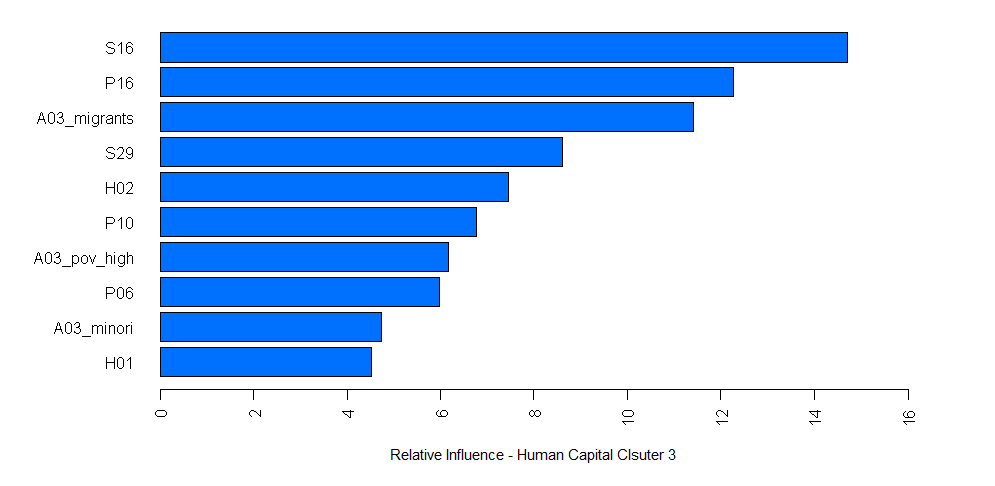


| **Variable Code** | **Variable Name** |
| --- | --- |
| S16 | Functioning and equitable water services |
| P16 | Energy sources |
| A03_migrants | Percentage of the population considered migrants |
| S29 | National policy & plan for forecasting ability |
| H02 | Personal safety |
| P10 | Communication infrastructure |
| A03_pov_high | Percentage of high income population |
| P06 | Individual (Household) Flood Vulnerability Management |
| A03_minori | Percentage of the population considered minorities |
| H01 | Flood protective behavior and knowledge |


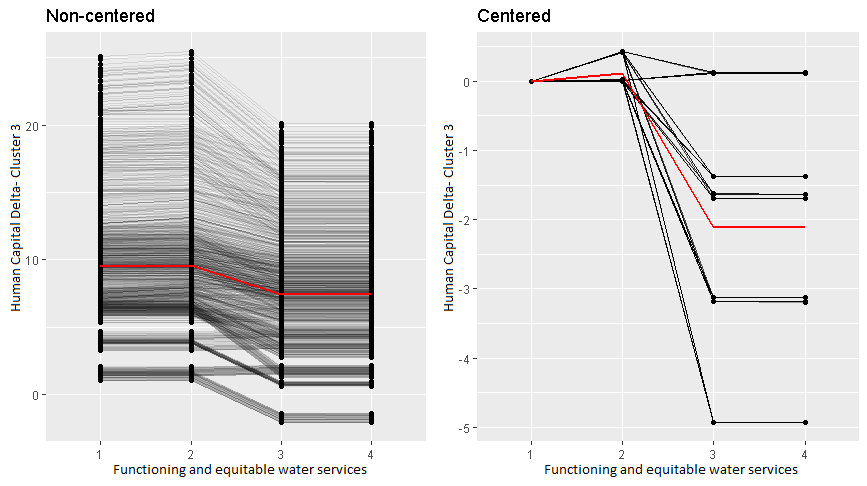


Note: The largest drop is between C and B.

**
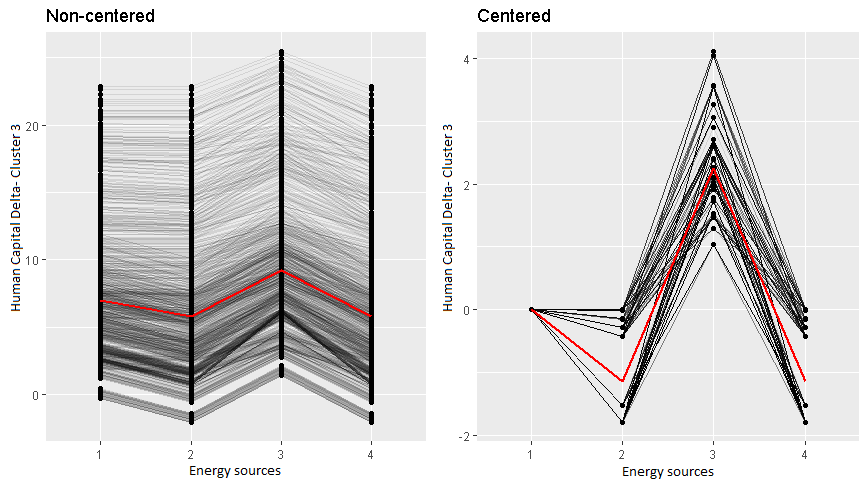
**

Note: Relationship between the Human capital delta and P16 Energy sources. Largest increase in Human Capital Delta is when P16 has a grade of 3 (B) that is “Energy sources are impacted but continue to be adequately operational”.


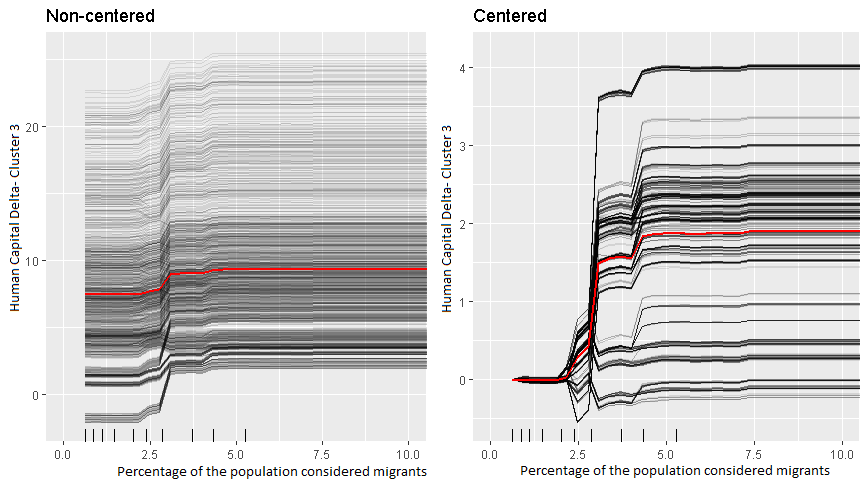


Note: There is a positive relation between the Human Capital Delta in cluster 3 and the percentage of the population considered migrants.

**Cluster 3 (iii) Natural Capital:**


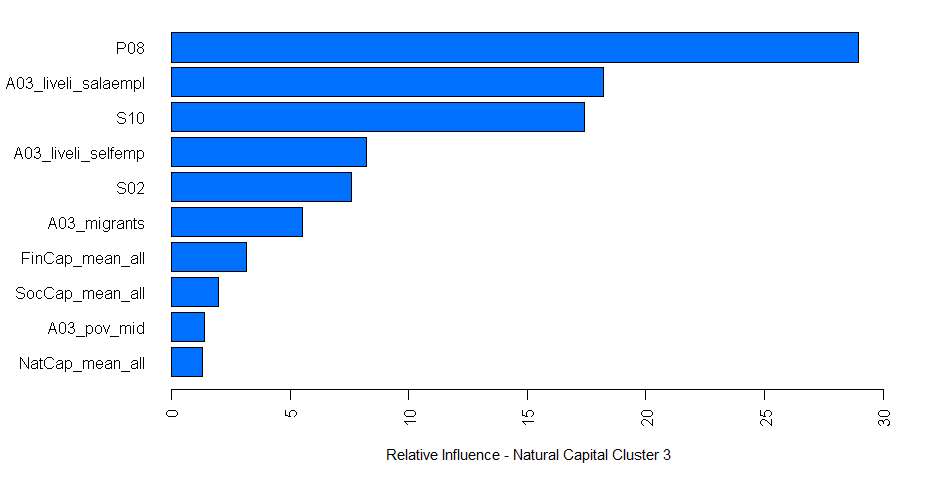


| **Variable Code** | **Variable Name** |
| --- | --- |
| P08 | Basin Level Flood Controls |
| A03_liveli_salaempl | Percentage of the population reliant on income from salaried employment |
| S10 | Mutual assistance systems and safety nets |
| A03_liveli_selfemp | Percentage of the population reliant on income from self employment |
| S02 | Formal community emergency services integrate flood advice and management |
| A03_migrants | Percentage of the population considered migrants |
| FinCap_mean_all | Average of Financial Capital Variables |
| SocCap_mean_all | Average of Social Capital Variables |
| A03_pov_mid | Percentage of middle income population |
| Nat_cap_mean_all | Average of Natural Capital Variables |

Note: For cluster 3 the most informative variables for the Natural Capital Delta are P08 (Basin Level Flood Controls), The proportion of the population who are salaried employees, S10 (Mutual assistance systems and safety nets), Self employed population, S02 (Formal community emergency services integrate flood advice and management), the migrant population, and the level of the other Capitals (Financial, Social, Human and Physical further down the list) at the time of the BL.


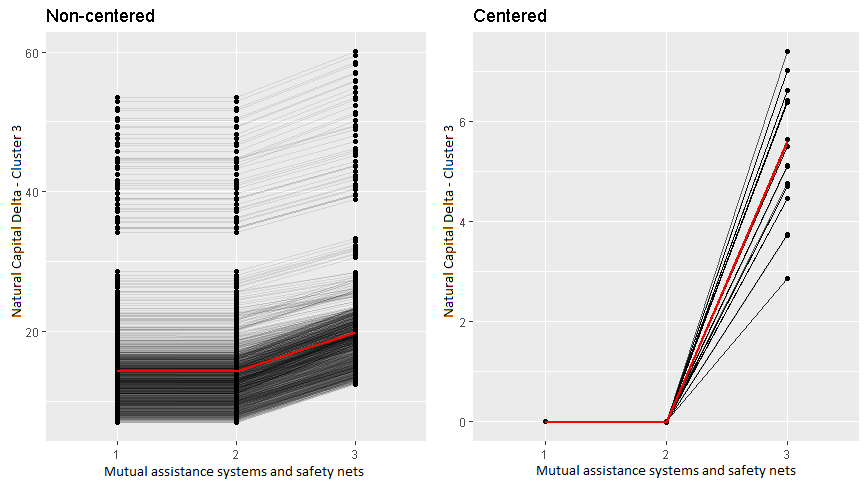


Note: Above the effect of P08 (Basin Level Flood Controls) are shown. A large increase in the Natural capital delta as the grade goes from a C (i.e. *IFRM at basin level is available but has no authority OR Limited IFRM at basin level exists but is inadequate to address flood risk*) to a B (i.e. *Integrated flood risk management (IFRM) exists or is being developed at basin level to improve flood risk*)


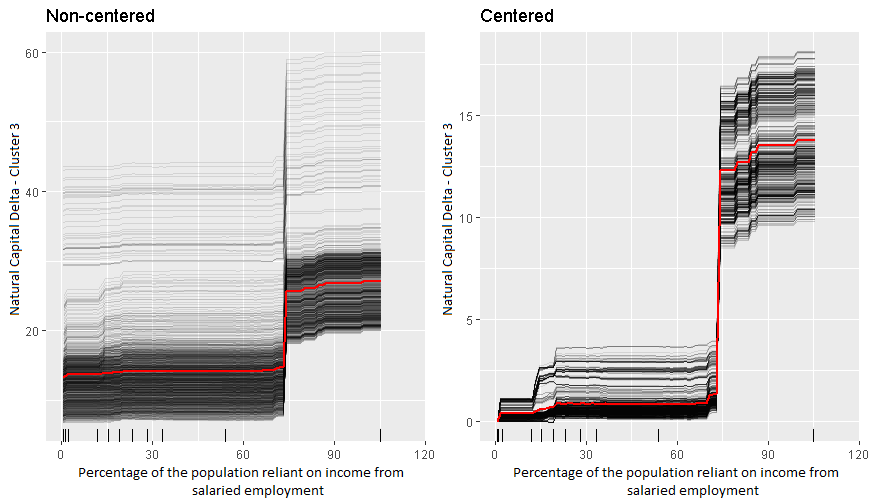


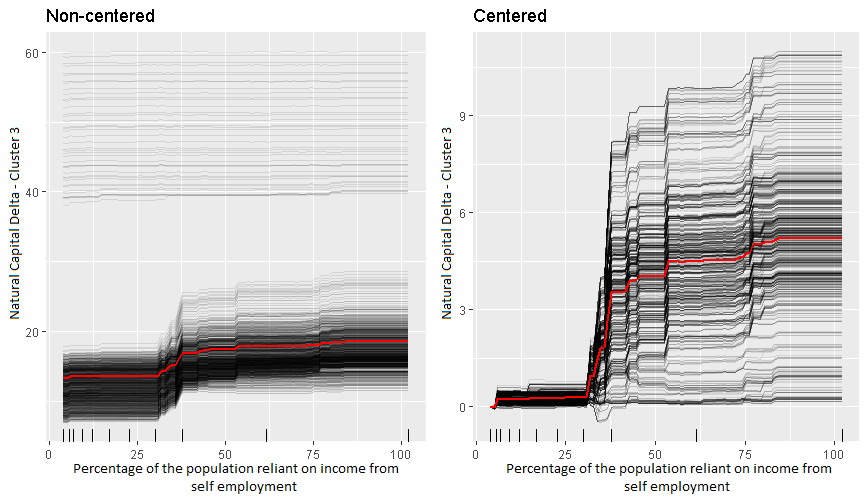


Note: A large jump at 75% & 30% respectively.


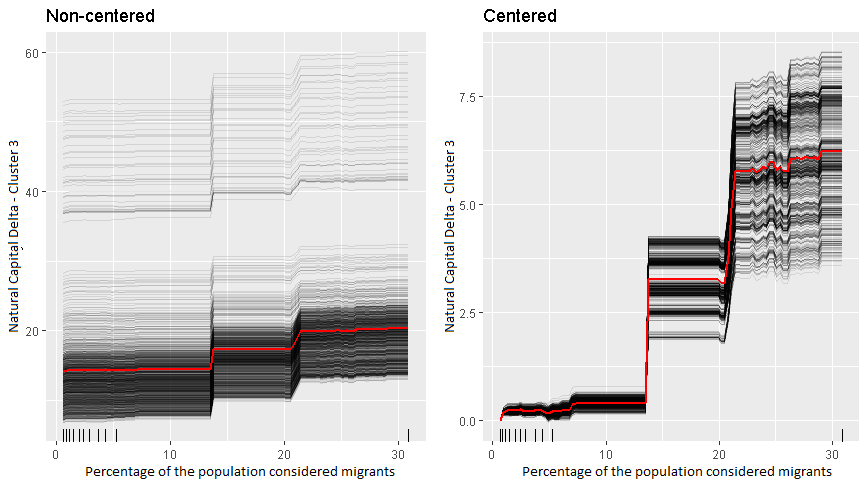


Note: Here we see the proportion of migrants in the population and their effects on the Natural Capital delta. We see jumps at 12% and 20%.

**Cluster 3 (iv) Physical Capital:**


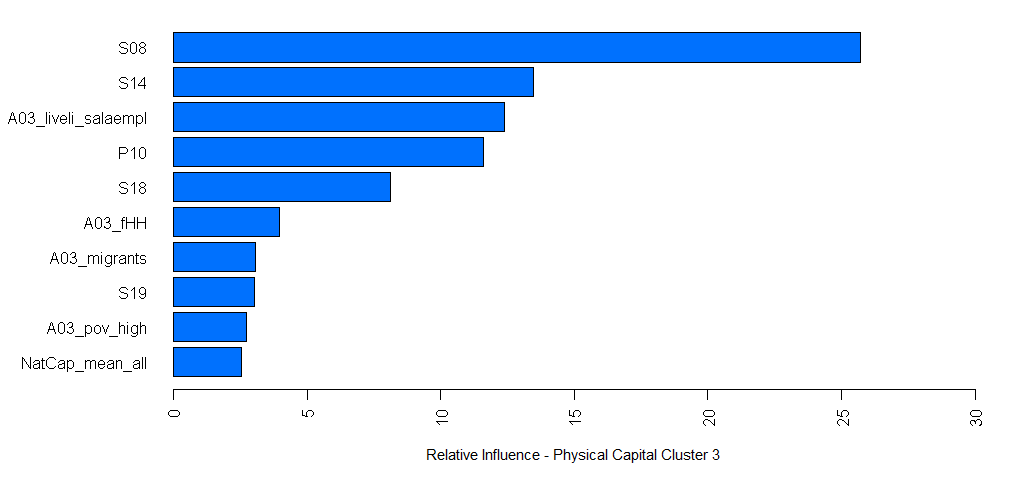


| **Variable Code** | **Variable Name** |
| --- | --- |
| S08 | Functioning and equitable education system |
| S14 | Functioning and equitable food supply systems |
| A03_liveli_salaempl | Percentage of the population reliant on income from salaried employment |
| P10 | Communication infrastructure |
| S18 | Functioning and equitable waste collection & disposal services |
| A03_fHH | Percentage of female headed households in the community |
| A03_migrants | Percentage of the population considered migrants |
| S19 | Strategy to maintain or quickly resume local waste collection & disposal services in the event of a flood |
| A03_pov_high | Percentage of high income population |
| NatCap_mean_all | Average of Natural Capital Variables |


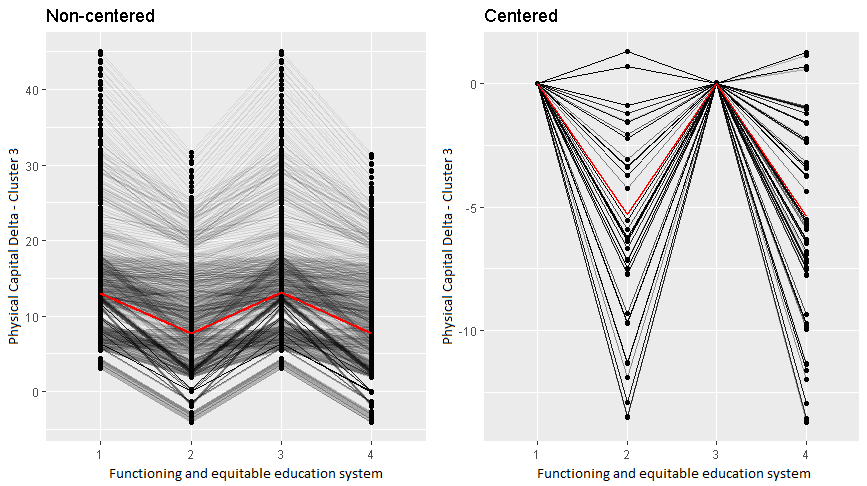


Note: The Individual conditional expectation of the Physical Capital Delta with respect to a S08-Functioning and Equitable education System is shown above. Similar to Cluster 1 there is a relation between the Physical Capital and a representation of the education of the community. As in cluster one we again hypothesize that in this case S08 acts as a proxy for the general infrastructure in the community.


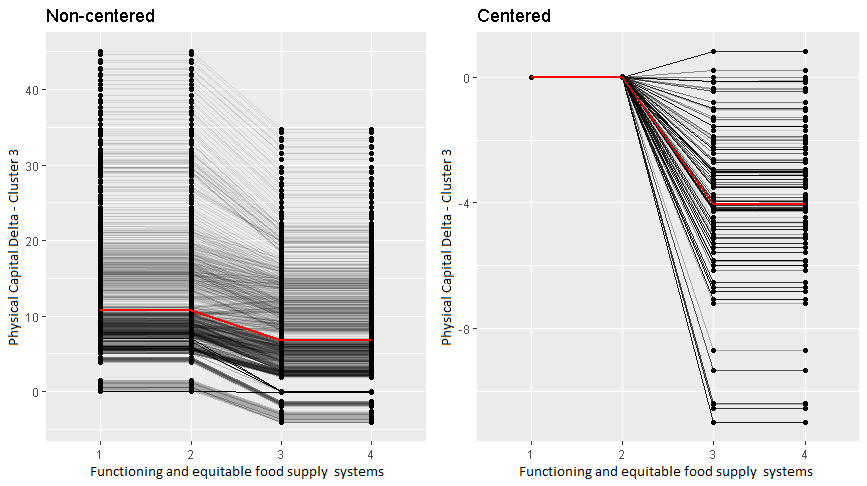


Note: Relation between Physical Capital delta and S14- Functioning and equitable food supply systems. We see a negative relation with the largest decrease found when S08 goes from a 2(C) to a 3(B)


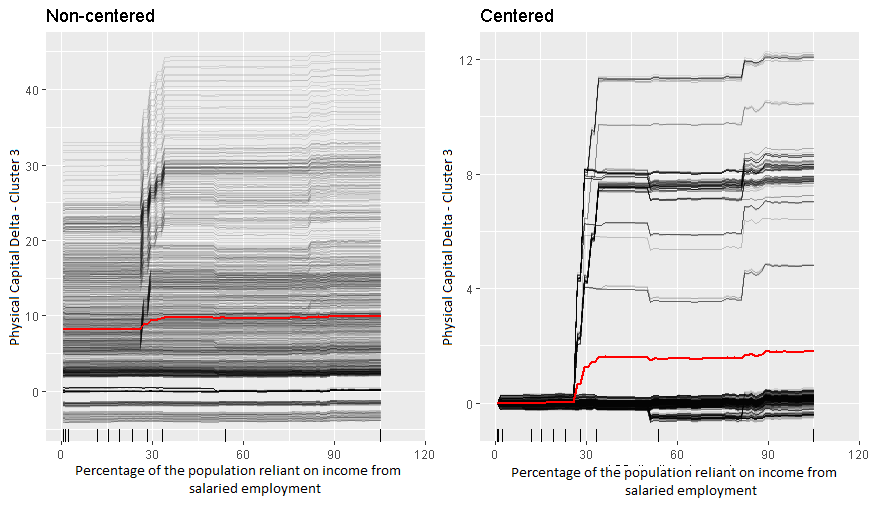


Note: Relation between the Physical Capital Delta and the percentage of the population which are salaried employees.

**Cluster 3 (v) Social Capital:**


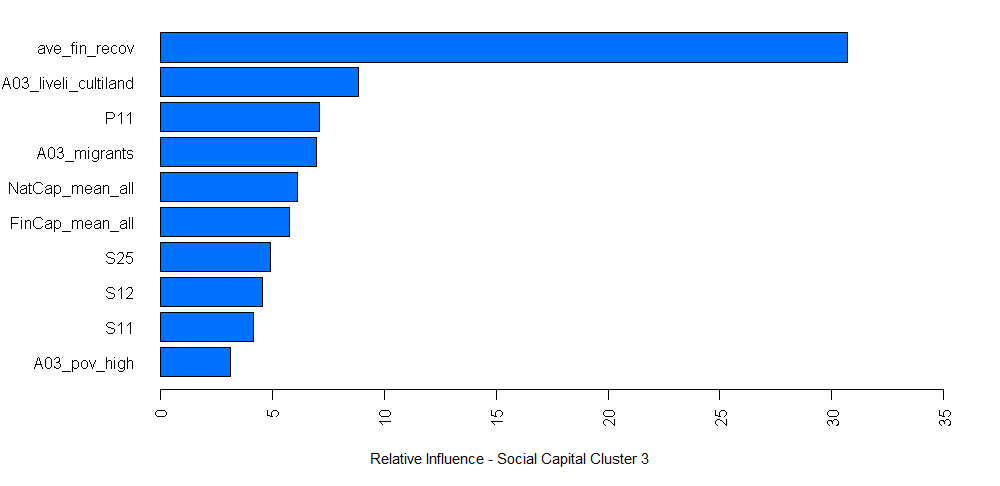


| **Variable Code** | **Variable Name** |
| --- | --- |
| ave_fin_recov | Average Financial Recovery |
| A03_liveli_cultiland | Percentage of the population reliant on income from cultivating their own land |
| P11 | Lifelines infrastructure |
| A03_migrants | Percentage of the population considered migrants |
| NatCap_mean_all | Average of Natural Capital Variables |
| FinCap_mean_all | Average of Financial Capital Variables |
| S25 | Culture for community information sharing |
| S12 | Appropriate and equitable access to mobility |
| S11 | Social norms and security of assets |
| A03_pov_high | Percentage of high income population |

Note: For the Social Capital delta in cluster 3 the most informative variables are financial recovery, proportion of the population who cultivate land, P11 (Lifelines infrastructure) the migrant population, the average of financial and natural capitals at the time of the Baseline.


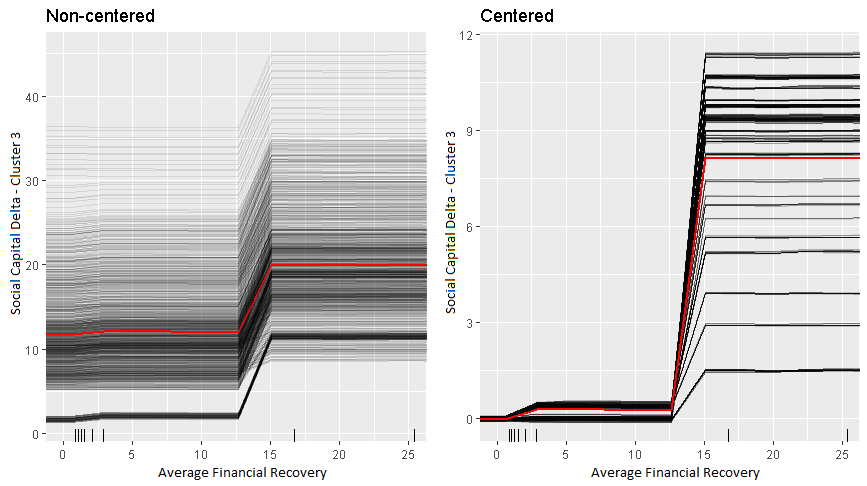


Note: Above the effect of average financial recovery on the Social Capital delta is shown. There is a jump at ~12.5% on average ~8.5 points


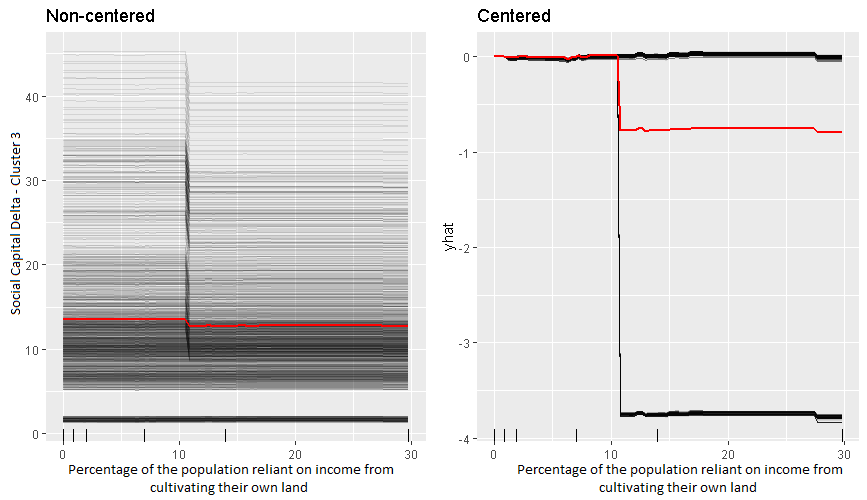


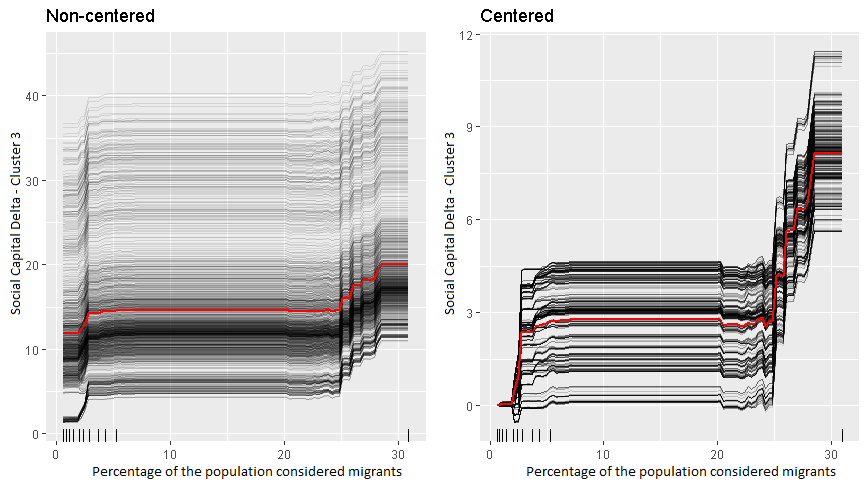


Note: This graph shows the migrant population. There is a jump at ~2.5% and again at 25%.


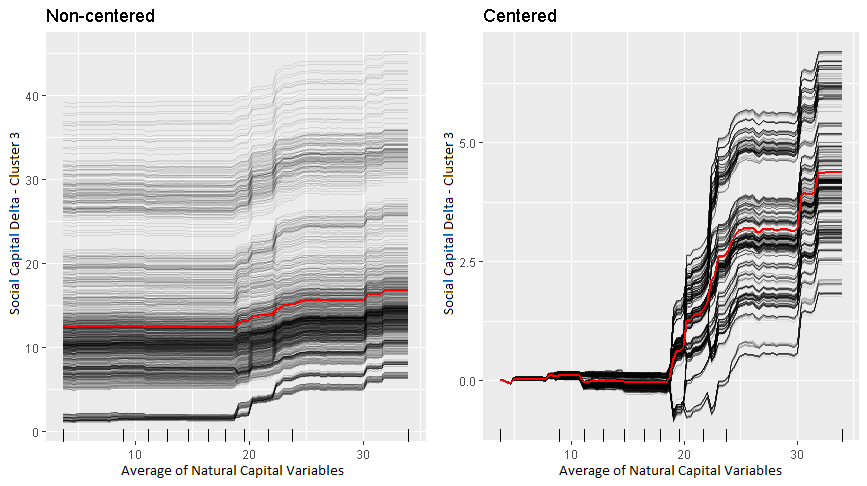


Note: A positive effect of the mean natural capital at the time of the Baseline with the Social capital increase


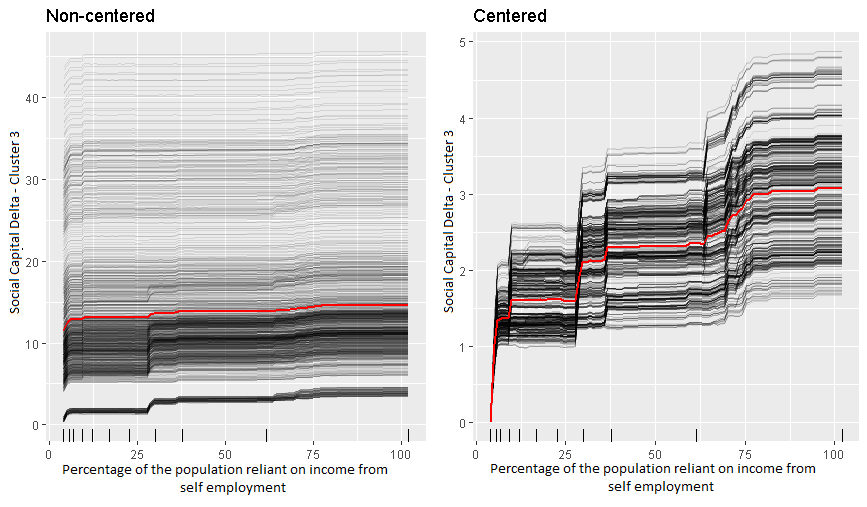


Note: As the proportion of self-employed people increases so does the delta of the social capital.


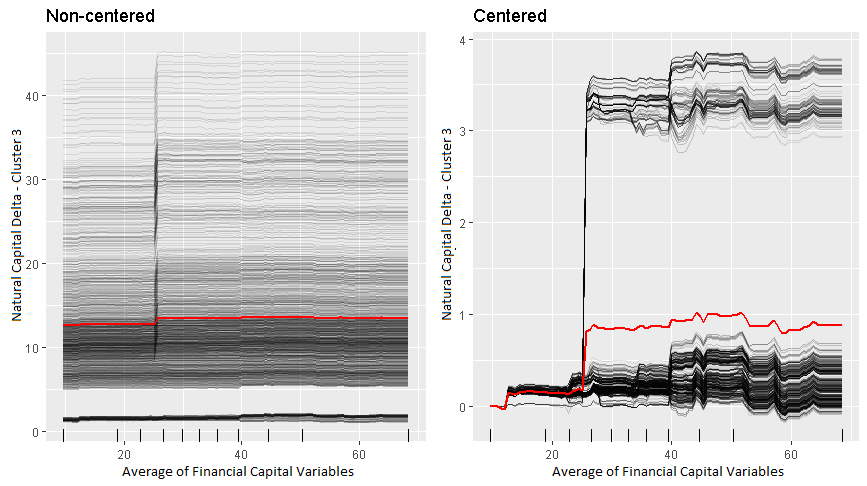


Note: This graph shows the effect of the Average Financial capital at BL and the Social delta. There seem to be two subgroups of communities within the cluster. One for which average Financial capital is at ~24 points and experiences a large jump in Social Capital delta and the other (larger) group for which Financial Capital does not play a major role for the Social Capital increase.


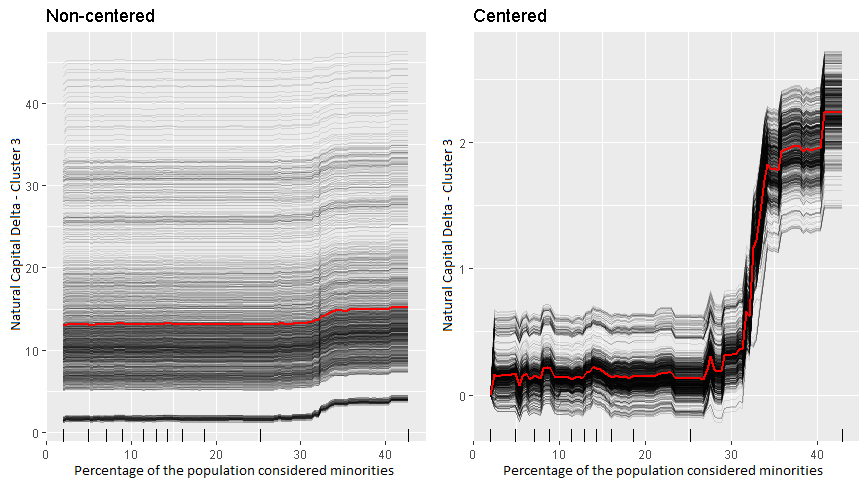


Note: This chart shows that if a community’s minority population is at 30% or more then they experience a large jump in Social Capital delta.
